# Associations Between Second-Language Proficiency and Executive Functions in Autistic and Neurotypical Children

**DOI:** 10.1162/OPMI.a.349

**Published:** 2026-04-17

**Authors:** Franziska Baumeister, Pauline Wolfer, Elisabet Vila Borrellas, Ehsan Solaimani, Inge-Marie Eigsti, Stephanie Durrleman

**Affiliations:** Autism, Bilingualism, Cognitive and Communicative Development Research Group (ABCCD), Faculty of Science and Medicine, University of Fribourg, Fribourg, Switzerland; Department of Translation and Language Sciences, Pompeu Fabra University, Barcelona, Spain; Department of Catalan Philology and General Linguistics, University of Barcelona, Barcelona, Spain; Department of Language and Linguistic Science, University of York, York, UK; Department of Psychological Sciences, University of Connecticut, Storrs, CT, USA

**Keywords:** attention, inhibitory control, working memory, switching, Bayesian multilevel modeling, entropy, flexibility

## Abstract

Bilingualism is a complex experience that may enhance executive functions (EF). This potential benefit could be particularly relevant for autistic children, given that they tend to present EF difficulties. Some studies find bilingualism benefits in attention, inhibitory control, cognitive flexibility, and memory, in neurotypical and autistic children while others do not. These inconsistencies may arise from oversimplifying the operationalization of bilingualism, obscuring the mechanisms underlying its effects on EF. This study used continuous measures to operationalize bilingualism and test its links with EF. Bayesian multilevel modeling showed that higher second language proficiency in 168 autistic (*M* = 8;3) and 262 neurotypical (*M* = 7;8) children was associated with better attention and working memory in neurotypical children and better attention, short-term memory, working memory, and shifting abilities in autistic children.

## INTRODUCTION

The experience of hearing or speaking multiple languages is associated with several benefits. These include engaging with individuals across backgrounds, occupational advantages, and exposure to other cultures (de Bruin et al., 2021). Bilingualism can also support communication within the family and foster positive identity formation, particularly for children whose families speak a language other than the societal language (Hampton et al., 2017; Howard et al., 2021; Müller et al., 2020).

Bilingualism has also been claimed to positively impact executive functions (EF; Bialystok, 2024), though findings are inconsistent (Paap, 2019). The potential impact of bilingualism on EF is particularly relevant for individuals with autism spectrum disorder (ASD), who experience EF impairments (Craig et al., 2016). Research to date on bilingualism effects on EF in autistic children is inconsistent (e.g., Gonzalez-Barrero & Nadig, 2019), reflecting methodological differences, including how EF is measured (e.g., parent-rated scales vs. game-based tasks), as well as an oversimplified definition of bilingualism (Romero et al., 2024). Reliance on a simple monolingual versus bilingual dichotomy masks critical variation in the complexity of language experience, including quality, quantity, and proficiency in each language (Grundy, 2020).

In this study, we explicitly address this issue by operationalizing bilingualism as a set of continuous, theory-driven dimensions. This approach allows us to examine how continuous dimensions (e.g., frequency of switching between languages, amount of exposure to and use of different languages, proficiency in the second language) of bilingual experience, and not bilingual status, relate to EF across autistic and neurotypical children. This dimensional approach is supported by recent theoretical and methodological critiques of binary bilingual classifications, which risk oversimplifying a highly variable experience (de Bruin, 2019; Kremin & Byers-Heinlein, 2021). A continuous modeling of bilingualism better reflects real-world language use and allows for testing specific hypotheses about how distinct facets of bilingual experience relate to cognitive development (de Bruin et al., 2021; Marian & Hayakawa, 2021; Rothman et al., 2023).

This study adds to the literature by examining the effects of bilingual experiences, conceptualized in a theoretically-driven and nuanced way along multiple dimensions, on core EF abilities of *attention*, *inhibitory control*, *short-term*- (STM) and *working memory* (WM), and *shifting* and *switching*. Using a multilevel Bayesian approach, we operationalized different theoretical accounts of the influence of bilingualism on EF, examining the role of sensitive bilingual predictors (*balance of use of different languages*, *code-switching frequency*, *richness of exposure to and use of the second-best language*, *proficiency in the second-best language*) in English, French, German, Italian and Spanish autistic and neurotypical children.

### Executive Functions

Executive functions are a set of cognitive processes essential for goal-oriented tasks, including planning, reasoning, attention regulation, and problem-solving (Diamond, 2013). EF are particularly needed when automatic behavior is insufficient to achieve a specific goal, enabling individuals to adapt to novel situations and manage complex life challenges (Collins & Koechlin, 2012). Early theoretical accounts of EF focused on attentional control (Posner, 1980), while more recent approaches divide EF into three core components: inhibitory control, WM, and cognitive flexibility, which work together to support higher-order functions like reasoning, planning and fluid intelligence (Diamond, 2013; Miyake et al., 2000). Across these components, *attention* is crucial for regulating environmental cues that guide information processing (Rueda et al., 2023; Rueda & Posner, 2013). *Inhibitory control* allows individuals to suppress impulsive responses, enabling goal-directed behavior. It interacts closely with WM to support selective attention and emotional regulation (Diamond, 2013). *Working memory* refers to the ability to hold information in STM and manipulate information that is no longer present. WM is essential for tasks like problem-solving and learning, and it interacts with inhibitory control to help manage behavior and prevent distractions (Diamond, 2013). Although STM and WM are sometimes separated on the basis of whether information must be manipulated, it is now widely recognized that they reflect a continuum of executive involvement (Baddeley et al., 2020). Even simple span paradigms such as forward visuo-spatial recall often place demands on controlled attention and goal maintenance and are frequently used as WM measures in developmental and bilingualism studies (e.g., Morales et al., 2013).

*Cognitive flexibility* is the ability to shift from one task to another or to switch frequently between tasks or perspectives in response to changing conditions. It builds on inhibitory control and WM, and is crucial for adapting to novel situations (Diamond, 2013).

EF develop sequentially, with basic components like inhibitory control and WM emerging in early childhood. Cognitive flexibility and higher-order functions, such as reasoning and problem-solving, develop later, continuing into adolescence. These processes become increasingly integrated, allowing for complex and adaptive behavior as individuals mature (Cristofori et al., 2019).

### Executive Functions in Autism Spectrum Disorder

Autism spectrum disorder (ASD) is a neurodevelopmental disorder characterized by persistent difficulties in social interaction and communication, repetitive and restricted behaviors (World Health Organization, 2022). A substantial body of research has shown that autistic children struggle with core EF abilities, such as WM, cognitive flexibility, and inhibitory control, which can contribute to broader difficulties in higher-order EF. A meta-analysis investigating WM differences between autistic and NT individuals analyzed data from 28 studies, including 15 studies with autistic children younger than 12 years (Wang et al., 2017). The meta-analysis demonstrated weaker performance among autistic participants, and suggested that spatial WM was more affected than verbal WM; age and IQ did not influence the severity of the difficulty. This specific finding for WM was supported further by a larger meta-analysis including 235 studies targeting all core EF components (Demetriou et al., 2018). Autistic children ages 12 and younger had weaker abilities than an age-matched NT control group in all domains, with no differences in effect sizes across domains (Geurts et al., 2009; Lage et al., 2024; Lai et al., 2017; Van Eylen et al., 2011).

A recent meta-analysis of cognitive flexibility in autistic children (Lage et al., 2024) reported a robust, moderate-to-large impairment (pooled Hedges g ≈ 0.6–0.8) that generalized across both set-shifting and task-switching paradigms. Crucially, the magnitude of this effect was task- and sample-dependent (Geurts et al., 2009; Van Eylen et al., 2011). Parallel meta-analytic evidence shows that autistic children also display moderate difficulties with inhibitory control (g ≈ 0.5–0.6), reinforcing the picture of a broader executive-function profile of impairment (Lai et al., 2017). Fewer studies have investigated attentional abilities in autism. A series of studies found weaker performance in autistic children (e.g., Berenguer et al., 2018), although an older study with 23 autistic children did not report a difference (Pascualvaca et al., 1998). These mixed findings are further underscored by Van Eylen et al. (2011), who highlighted inconsistencies in the literature on cognitive flexibility in ASD, both within the same task and across different paradigms. Notably, while deficits are often reported in naturalistic contexts, clinical and experimental settings yield more heterogeneous results (Geurts et al., 2009).

Given the difficulties that children with ASD present in EF, the question arises whether bilingualism, which arguably boosts EF in neurotypical individuals (Bialystok, 2024), may serve to mitigate EF difficulties in autistic children.

### Effects of Bilingualism on Executive Functions

The activation of multiple language systems in the brain, even in contexts requiring only one language, suggests that bilinguals need to manage competition between their languages (Kroll & Bialystok, 2013). The experience of linguistic interference could strengthen EF abilities by providing enhanced experience with managing multiple tasks, switching between tasks, and inhibiting distractions. Several accounts have been proposed to shed light on how bilingualism affects EF. The ***task switching hypothesis*** posits that bilinguals who have experience switching frequently between languages will perform better on tasks requiring switching skills (Prior & Gollan, 2011). The ***inhibitory control hypothesis*** (Green, 1998) proposes that bilinguals engage in frequent inhibitory control to suppress interference from non-target languages (Bialystok et al., 2004). The ***adaptive control hypothesis*** (ACH; Green & Abutalebi, 2013) extends the *inhibitory control hypothesis* by incorporating broader language control processes, including monitoring, conflict resolution, and shifting, across different interactional contexts. This account suggests that bilinguals’ EF skills are shaped by the interactional context of language use: single-language, dual-language, and dense code-switching. In the single-language context, bilinguals use one language exclusively, requiring goal-directed behavior and suppression of interference from the non-relevant language. The dual-language context involves frequent language switches at sentence boundaries, which is thought to impose higher demands on interference control and salient cue detection; talkers must remain vigilant and responsive to environmental or conversational signals indicating a language switch. Finally, in the dense code-switching context, speakers switch languages *within* utterances, which is theorized to enhance opportunistic planning and flexibility with lesser demands on inhibitory control. Other accounts focus on EF as a whole, rather than on its specific subcomponents. For example, Bialystok situates the effects of bilingualism within the role of ***executive attention*** and general adaptational processes (Bialystok, 2017). According to this account, bilinguals’ better performance on complex cognitive tasks is due to a domain-general system in which attention allows for better cognitive control. As these accounts were formulated in the context of neurotypical development, studies of the impact of bilingualism on EF in autistic children have been limited to date, despite the far-reaching implications for quality of life.

While these accounts build on similar rationales, and are therefore not mutually exclusive, each emphasizes distinct aspects of the cognitive demands associated with bilingualism (Bialystok, 2017). The ICH focuses on the need to *suppress* non-target languages; the ACH highlights the role of interactional contexts in shaping *control mechanisms*; and the EAA considers the effect of bilingualism on *domain-general attentional systems*. Together, these accounts offer complementary perspectives on how bilingual experience may shape EF.

#### Evidence for the Effects of Bilingualism on Executive Functions in Neurotypical and Autistic Children.

Research on the effects of bilingualism on EF in neurotypical children is extensive, with ongoing debate about the bilingual advantage (see Antoniou et al., 2016 and de Bruin et al., 2021 for an overview). A meta-analysis of 143 studies found a small advantage for switching, inhibition, and monitoring in bilingual neurotypical children ages 0–18 years, controlling for publication bias (Gunnerud et al., 2020). Yurtsever et al. (2023) observed that neurotypical bilinguals outperformed monolinguals more often than chance. In contrast, a meta-analysis of EF in 149 studies of neurotypical bilingual versus neurotypical monolingual children ages 3 to 17 years found a small bilingual advantage, which became negligible after adjusting for publication bias (Lowe et al., 2021). Similarly, Paap et al. (2025) concluded that neither cognitive training nor bilingualism enhances general cognitive abilities when accounting for publication bias and study quality. They argue that null results from both fields reinforce each other, suggesting no special cognitive benefits from bilingualism beyond social and communicative advantages. Significant variability across studies in how bilingualism was measured and described suggests a need for more sensitive measures that can capture the factors driving any bilingual advantage. Below, we review research studying the relationship between bilingualism and specific EF components.

#### Inhibitory Control.

Overall, research on inhibitory control has produced mixed results. Many studies have demonstrated that neurotypical bilingual children perform better than neurotypical monolinguals (e.g., Carlson & Meltzoff, 2008), while others found no difference (e.g., Morton & Harper, 2007). In contrast, research on the impact of bilingualism on EF in autistic children has been limited. Li et al. (2017) examined the performance of 67 children, including 13 bilingual autistic children (mean age: 9;2), and 19 monolingual autistic children (mean age: 8;4) on the Stroop task. Results indicated faster reaction times in bilingual compared to monolingual autistic participants, suggesting enhanced inhibitory control. Sharaan et al. (2021) investigated the attention abilities of 27 bilingual autistic children and 10 monolingual autistic children, aged five to 12 years, matched on age, nonverbal IQ, and socioeconomic status. All children had a nonverbal IQ within the ‘intellectually average’ range or above. The study found that bilingual autistic children had fewer false starts on sustained attention tasks, indicating better self-regulation and inhibitory control than monolingual peers. Similarly, Gonzalez-Barrero and Nadig (2019) reported a bilingual advantage over monolinguals in tasks requiring conflict monitoring and inhibitory control, using the Go/No-Go task. They examined 40 children aged 6–9 years (10 bilingual autistic, 10 monolingual autistic, 10 bilingual neurotypical, and 10 monolingual neurotypical) with a nonverbal IQ above 80, matched on maternal education level and gender. Romero et al. (2024) tested inhibitory control skills in 53 children aged 7–12 years, using a parental questionnaire. They reported that bilingual autistic children had stronger inhibition skills compared to their monolingual peers. Furthermore, Montgomery et al. (2022) reported that increased bilingual exposure was associated with better prepotent response inhibition in 38 autistic participants between 5 and 13 years, with no advantage on a flanker task. In contrast, Gonzalez-Barrero and Nadig (2019) observed no differences for response inhibition, and Sharaan et al. (2021) found no bilingual advantage in the Simon task compared to monolingual peers.

#### Working Memory.

Studies assessing WM have yielded inconsistent results. Several studies reported a bilingual WM advantage in neurotypical children, particularly on tasks with greater executive demands. For instance, Morales et al. (2013) reported that NT bilinguals outperformed monolinguals in WM tasks when additional executive demands were present. However, other studies reported no difference on simpler WM tasks (e.g., Blom et al., 2014). Studies of WM in autism are similarly inconclusive. Some studies, like the ones by Gonzalez-Barrero and Nadig (2019) and by Li et al. (2017), found no bilingual advantage over monolinguals on WM tasks such as the number repetition task or on the parent-report *BRIEF* WM scale. In contrast, Peristeri et al. (2021) showed better performance by bilingual compared to monolingual autistic children in a backward digit span task.

#### Flexibility.

Studies assessing cognitive flexibility using paradigms like the *Dimensional Change Card Sorting task* (DCCS; Zelazo et al., 1996) have generally shown more positive effects of bilingualism in NT children (e.g., Carlson & Meltzoff, 2008). However, other studies did not report an advantage (e.g., Filippi et al., 2022). Evidence on cognitive flexibility is clearer in autistic children, with several studies reporting a bilingual advantage. Gonzalez-Barrero and Nadig (2019) found that bilingual autistic children outperformed monolingual peers on set-shifting tasks, such as the border version of the *DCCS*, which requires cognitive flexibility. Similarly, bilingual autistic children performed better in a global-local task than their monolingual peers. Romero et al. (2024) reported better shifting abilities in multilinguals compared to monolinguals, as assessed with a parental questionnaire. In contrast, Sharaan et al. (2021) found no bilingual advantage.

#### Attention.

Research on NT and autistic children has also shown mixed outcomes. Some studies, like Yang (2008), reported better attentional control for neurotypical bilinguals. However, other studies reported no bilingual advantage (Nguyen et al., 2024). Sharaan et al. (2021) found that bilingual children with ASD performed better on sustained attention tasks, with fewer lapses in attention, compared to monolingual children. However, other studies, such as those by Gonzalez-Barrero and Nadig (2019) and Li et al. (2017), found no attentional differences between bilingual and monolingual children with ASD.

#### Summary.

Research on bilingualism and EF in both neurotypical and autistic children presents a mixed picture. There is strong evidence for an advantage in inhibitory control and cognitive flexibility in bilingual neurotypical children, compared to monolingual peers. In comparison to monolingual autistic children, there is also strong evidence of an advantage in cognitive flexibility and conflict monitoring in bilingual autistic children, and more limited evidence of a bilingual advantage in WM. However, studies on bilingualism in autism have often relied on small samples. Additionally, across these studies, bilingualism has been operationalized as a category (mono- versus bilingual). However, bilingualism consists of factors that vary along a continuum (Grundy, 2020; Luk & Bialystok, 2013) including: the *quantity* of language exposure and use; the *age* of first exposure to different languages; the *quality* of the exposure and use; the speaker’s *proficiency* in each language; and *switching* habits (de Bruin et al., 2021). One way to capture this bilingual heterogeneity, aligned with the ACH, is to create additional categories of single-language users, dual-language users and dense code-switching users (e.g., Beatty-Martínez et al., 2020). Another approach, adopted in the current study, is to use *entropy* (Gullifer & Titone, 2020) as a means of capturing the diversity of language use across communicative contexts; higher scores indicate greater diversity in the use of languages and less predictability (Gullifer et al., 2018).

In sum, while prior research offers valuable insights into the potential relationship between bilingualism and EF, especially in autistic children, it is limited by three major issues: (1) small and often under-powered samples, particularly in studies involving autistic participants; (2) inconsistent operationalization of bilingualism, with most studies relying on binary classifications (monolingual vs. bilingual); and (3) a lack of alignment between study design and theoretical accounts of EF development.

The current study addresses these limitations by drawing on a large and diverse sample of both autistic and neurotypical children (*N* = 430), using continuous, theory-driven measures of bilingualism, and explicitly testing predictions derived from three major EF accounts: the inhibitory control hypothesis, the adaptive control hypothesis, and the executive attention account. This approach allows for a more precise and generalizable understanding of how specific bilingual experiences relate to EF development across neurodiverse populations.

### Present Study

The present study tests the effects of language experience on EF in children with and without autism, measuring diverse language experiences of up to three languages in a continuous manner. We assessed EF in a large cohort of autistic and neurotypical children aged 3–12, a key developmental period during which core EF emerge and develop in both populations (neurotypical: Best & Miller, 2010; Diamond, 2013; ASD: Demetriou et al., 2018; Wang et al., 2017). We evaluated the degree of bilingualism using a detailed questionnaire. The present study first tests specific hypotheses derived from the theoretical accounts, followed by a more confirmatory investigation into the impact of bilingualism on EF.

The study was designed to test three current accounts of EF in bilingualism: The (1) inhibitory control hypothesis, (2) the adaptive control hypothesis, and (3) the executive attention account. Here we give an outline of the predictions of each account in turn, and explain how they relate to different aspects of bilingualism.

According to (1) the inhibitory control hypothesis (ICH), the need for frequent inhibition of irrelevant languages and monitoring of the surroundings would lead to better non-verbal inhibitory control. This is based on the idea that the cognitive processes involved in inhibiting a non-target language rely on domain-general inhibitory control mechanisms, which are also involved in suppressing irrelevant information in non-verbal contexts (e.g., conflict tasks like the Simon task). Thus, practice with language suppression is thought to enhance inhibitory control more broadly, beyond the language domain. The need for “frequent inhibition of irrelevant languages” was operationalized in this study as the *balance of cumulative use of different languages* throughout a participant’s life. The higher the balance, the more often the individual would need to inhibit other languages. This balance reflects the level of cognitive control required to manage multiple languages in everyday life and therefore serves as a meaningful proxy for the inhibitory demands proposed by the ICH. The ICH would be supported if we found that a more balanced usage was associated with better inhibitory control (reaction times or accuracy on incongruent items on the Simon task).

According to (2) the adaptive control hypothesis (ACH; Green & Abutalebi, 2013), individuals’ bilingual language use can be characterized by three interactional contexts: single-language, dual-language, and dense code-switching. In the current study, we operationalized these contexts using two variables: (1) *balance of language use within contexts*, and (2) *frequency of code-switching*. A higher balance of language use within contexts (i.e., more equal use of both languages in the same settings such as home, school, or community) reflects a dual-language context that requires increased continuous engagement of both language systems and frequent task engagement, disengagement, and shifting. In contrast, a lower balance of use indicates a more predominantly single-language context, characterized by sustained inhibition of the non-target language. Frequency of code-switching differentiates dual-language contexts (lower frequency of code-switching between utterances) from dense code-switching contexts (high frequency of intra-sentential switching), which are thought to rely on cooperative control with reduced inhibitory demands. Based on the ACH, we predicted that greater balance of use within contexts (reflecting stronger dual-language engagement) would be associated with better inhibitory control and shifting/switching performance, as these contexts involve frequent selection and disengagement between competing linguistic systems. In contrast, higher frequency of code-switching was expected to be associated with reduced inhibitory control demands as both languages remain jointly active and do not require suppression of a non-target language.

Although the Adaptive Control Hypothesis does not generate strong directional predictions for WM or attention as separable EF components, it does imply sustained engagement of attentional resources and maintenance of task goals during bilingual language use. For this reason, associations between ACH-related bilingualism variables and WM or attention were examined on an exploratory basis.

Lastly, according to (3) the executive attention account (EAA), the effects of bilingualism are expected to lead to general improvement across multiple EF domains, due to enhanced attentional control mechanisms. This account draws on theoretical perspectives that view executive attention either as a central component of executive function or as a foundational process that supports other EF domains (e.g., Diamond, 2013). It is based on the idea that bilingual individuals develop the ability to selectively focus on task-relevant information, inhibit interference, and manage competing representations in working memory, abilities that are proposed to underpin broader cognitive control (Engle & Kane, 2004). Bialystok (2017) situates the effects of bilingualism within this attentional control framework, suggesting that bilinguals’ enhanced performance on complex cognitive tasks reflects adaptations in a domain-general system of attention. As the EAA does not specify which particular features of bilingual experience might drive these adaptations, we operationalized this hypothesis through three alternative variables that are all likely to place sustained demands on attentional control. These included: (a) the *balance of cumulative use of different languages*, capturing the extent to which both languages are used regularly and therefore require ongoing attentional regulation (a quantitative dimension), (b) the *richness of the exposure to and use of the second-best language* (a qualitative dimension), and (c) the *proficiency in the second-best language*, reflecting the idea that higher proficiency in both languages may result from, and further demand, efficient coordination of competing language systems. This account would be supported if these bilingualism-related variables were associated with better attention, inhibitory control, short-term memory, WM, and cognitive flexibility.

In light of the above, we examined the impact of bilingualism on various EF tasks among neurotypical and ASD children. We adopted a theory-driven approach, aligning specific bilingualism-related variables with the executive function domains most relevant to each theoretical account. Rather than testing all variables across all outcomes, predictors were selected based on their conceptual relevance to the mechanisms proposed by the aforementioned theoretical frameworks. This analytic structure responds to recent calls for more transparent and hypothesis-led research in bilingualism (de Bruin et al., 2021; Luk, 2023; Marian & Hayakawa, 2021; Rothman et al., 2023).

Specifically, we addressed the following research questions:

RQ1: Which of the accounts—inhibitory control, adaptive control, or executive attention—best accounts for the performance of autistic and neurotypical children across six EF domains (attention, inhibitory control, short-term memory, working memory, shifting, switching)?

RQ2: According to the “best” account (as determined in RQ1), what is the association between the bilingual experience and EF in both autistic and neurotypical children?

To investigate RQ1, for each of the six EF tasks we created a multilevel Bayesian model corresponding to the accounts (1), (2), and (3), respectively, and compared their goodness-of-fit. To address RQ2, we summarised the details of the best-fitting model which specifies how bilingualism experiences impacted EFs^1^.

## METHODS

### Participants

A total of 430 children ages 3 to 12 years participated in the study, as shown in Table 1; while many of the participating children completed all EF tasks, the sample varied by each specific EF component (see also Table S1.1 in the Supplementary Materials). Socioeconomic status was assessed as parental highest educational level (Hauser & Warren, 1997), which did not differ by group. Children, from Canada (*n* = 9), France (*n* = 80), Germany (*n* = 93), Spain (*n* = 43), Switzerland (*n* = 136), the United Kingdom (*n* = 41) and the United States (*n* = 28), were recruited through primary school contacts and autism associations, Facebook, psychologists, speech and language therapists, and the online recruitment platform “BuildClinical” in the US. All caregivers gave written informed consent, and participants were compensated with a gift card (35 CHF or 35€ or 60 CAD or 35 USD). All autistic participants had an official diagnosis of ASD from a psychiatrist or psychotherapist, supported by either the *Autism Diagnostic Observation Schedule-2^nd^ Edition* (*ADOS-2*; Lord et al., 2003) or another standardized ASD diagnosis tool such as the *Autism Diagnostic Interview-Revised* (ADI-R; Lord et al., 1994). Inclusion criteria for all participants were: (1) age between 3 and 12 years, to capture a key developmental window for EF development (Best & Miller, 2010; Demetriou et al., 2018), (2) enrollment in a larger project investigating bilingualism effects on cognitive and communicative development, and (3) availability of data on at least one EF measure to ensure inclusion in the primary analyses. Exclusion criteria for the NT group were parent-reported history of language or cognitive delays or impairments, or history of autism diagnosis. Participants completed the protocol in their most proficient language, which typically corresponded to the societal language of their environment. This approach was chosen to reduce potential confounds related to language comprehension. Information about the participants’ non-verbal IQ scores as measured with the *Raven’s-2* (Raven et al., 2018) can be found in Table 1 and Supplementary Materials S1.1, which also contain information about receptive vocabulary skills, assessed with the standardized *Peabody Picture Vocabulary, Test-4* (Dunn & Dunn, 2007) in the testing language (English, French, German, Italian or Spanish)^2^. Participants’ language experience characteristics are detailed in the Supplementary Materials S1.2, S1.3, and S1.4.

**Table 1.** Participant demographics.

	**ASD**	**Neurotypical**
***N***	168	262
**Age**, *M* (*SD*)	8;3 (2;3)	7;8 (2;5)
*Range*	3;1–12;0	3;2–11;11
**Gender** (*Diverse* | *Female* | *Male*)	1 | 36 | 131	1 | 133 | 128
**SES** / **Parent Educational Level**, *M* (*SD*)	4.2 (1.1)	4.6 (0.7)
*Range*	1–5	1–5
**Attention** (SWAN z-score), *M* (*SD*)	−0.6 (1.1)	0.6 (0.8)
*Range*	−2.9–2.7	−1.0–3.0
**Non-Verbal IQ** (Raven’s-2 IQ score), *M* (*SD*)	94.6 (14.8)	99.3 (13.1)
*Range*	66–133	66–133
**Receptive vocabulary** (PPVT-4 z-score), *M* (*SD*)	−1.0 (1.8)	0.4 (1.2)
*Range*	−4.0, 4.0	−4.0, 3.3

*Note*. ASD = autism spectrum disorder; NT = neurotypical; SD = Standard deviation; SES = socioeconomic status; *SWAN* = *Strengths and Weaknesses of Attention-Deficit/Hyperactivity Disorder Symptoms and Normal Behavior Scale* (Swanson et al., 2012); Parent educational level: 1 (elementary school) and 5 (university degree), *PPVT-4* = *Peabody Picture Vocabulary Test 4* (Dunn & Dunn, 2007).

### Materials

#### Procedure.

Children were tested individually by a trained experimenter in person over two sessions. All experimenters followed a standardized protocol and received systematic training to ensure consistency across sites. Tasks were screen-based and required non-verbal answers. Instructions and stimuli were adapted to the local language (i.e., English, French, German, Italian and Spanish) and pre-recorded to ensure consistency across experimenters and sessions. Scoring was automated to minimize rater-related variability.

#### Bilingual Language Experiences (Q-BEx).

Parents completed the *Quantifying Bilingual Experiences questionnaire* (*Q-BEx*; De Cat et al., 2022) as a continuous measure of a child’s language experiences (up to three languages). The *balance of cumulative use of different languages* (relevant to inhibitory control in the ICH, Account 1) in the form of an entropy score was calculated according to the formula, where *p*_*i*_ refers to the percentage of time of using language *i*:

$$H=-\sum_{i=1}^{n}p_{i}\cdot {\text{log}}_{2}\left(p_{i}\right)$$

(Shannon, 1948).

Higher entropy scores reflect a more balanced use (greater unpredictability about which language will be used). Possible scores range from 0 (monolingual experience) to 1.58 (very balanced use of three languages). To assess the *balance of use of different languages within contexts* (home, school, community, holidays), a measure of the interactional contexts in which speakers use different languages, relevant to the ACH and included in Account 2, we calculated separate entropy scores for each context, reflecting the degree of multilingual use within that setting. These four scores were then weighted based on the proportion of time participants reported spending in each context. A high balance score indicates frequent use of different languages within a specific context, aligning with dual or code-switching contexts. The *frequency of code-switching* was reported in the *Q-BEx*. To operationalize Bialystok’s executive attention account, the *richness of exposure to and use of the second-best language* was calculated as a composite percentage score of a series of questions about the quality of routine activities conducted in their second-best language (L2), such as frequency of reading (or being read to), writing, doing homework, school activities, screen-based activities, social interactions with friends, organized activities outside school. Finally, *proficiency in the second-best language* was calculated as a percentage composite of parental estimates of the child’s abilities in speaking, understanding, writing and reading in each language (range: 0–100%).

#### EF Tasks.

We selected four EF measures based on their suitability for use with both neurotypical and autistic children across a wide age range. Our selection criteria prioritized tasks that are non-verbal or minimally reliant on language, have demonstrated validity and reliability in developmental and clinical populations, and have been previously used in bilingualism research. This approach allowed us to construct a theory-aligned and developmentally appropriate battery covering core EF domains as defined in Diamond’s (2013) framework. A detailed description and rationale for each EF task is provided in the Methods section below.

#### Attention: SWAN Questionnaire.

The *Strengths and Weaknesses of Attention-Deficit/Hyperactivity Disorder Symptoms and Normal Behavior Scale* (*SWAN*; Swanson et al., 2012) is a nine-item parent questionnaire assessing symptoms of attention deficit hyperactivity disorder. The SWAN provides reliable dimensional ratings of attention and has been validated in samples with and without autism (Dupuis et al., 2022; Krakowski et al., 2020; Lakes et al., 2012; Swanson et al., 2012). Caregivers were asked to rate their child’s abilities in comparison to same-age peers on a 7-point Likert scale, ranging from ‘far below average’ (−3) to ‘far above average’ (3). Lower scores suggest poorer attention abilities. The summed scores were transformed to *z*-scores (*M* = 0, *SD* = 1).

#### Inhibitory Control: Simon Task.

The Simon task (Simon & Wolf, 1963) is a non-verbal task assessing conflict monitoring and conflict resolution processes, previously employed in studies of bilingual children and in autism research (Campbell, 2007; Simon & Wolf, 1963; Tse & Altarriba, 2014; see Romero & Uddin, 2021). Conflict monitoring involves detecting conflict and preparing specific subsequent actions, while conflict resolution includes inhibitory control (Lu & Proctor, 1995). A stimulus was presented in the upper left or right of the screen. Participants had to identify the stimulus based on color, as quickly as possible; a response to a blue stimulus required the participant to click a blue button on the bottom left, and a red stimulus required them to click a red button on the bottom right. For congruent items, the stimulus appeared above the same-colored button; for incongruent items, the stimulus appeared on the opposite side. To respond to incongruent items, participants had to suppress a prepotent response based on irrelevant information (location) and rely on task-relevant nonspatial information (color/shape). Correct responses to congruent and incongruent items were scored as 1, incorrect answers as 0.

#### Short-Term and Working Memory: Frog Matrices Task.

Visual short-term memory (STM) and visual working memory (WM) were assessed using the *Frog Matrices task* (*FMT*; Morales et al., 2013), administered on tablets. This non-verbal span paradigm has revealed bilingual advantages in childhood (Köder et al., 2022; Morales et al., 2013). A frog jumped between nine boxes in a 3x3 matrix. In the STM condition, participants had to recall the sequential order of the boxes the frog visited, starting with two positions, with a maximum of six positions. In the WM condition, participants performed the same task, but recalled the boxes in reverse order. Correct responses were scored as 1, incorrect answers as 0.

#### Shifting and Switching: Dimensional Change Card Sorting Task.

The *Dimensional Change Card Sorting task* (*DCCS*; Zelazo et al., 1996) assesses shifting, the ability to transition between two sorting rules, and switching, the ability to move back and forth between different rules and tasks. This task has been widely used with bilingual autistic and neurotypical children (Gonzalez-Barrero & Nadig, 2019; Zelazo et al., 1996). In this tablet-based task, participants saw a stimulus in the top center of the screen, a blue rabbit on the bottom left and a red boat on the bottom right. In Block 1, the participants had to sort six randomly presented stimuli (3 rabbits and 3 boats) according to their color (blue or red), for a total of six trials. In Block 2, participants had to sort the stimuli (rabbits, boats) according to their shape; they were informed of the sorting rule only once, at the start of the block. If a participant completed at least 5 trials correctly in each of Blocks 1 and 2, they were presented with Block 3 (switching condition); otherwise, the task was terminated. In the 16 trials of Block 3, stimuli were presented with a black border or no border. If there was a border, they were told to sort the stimuli according to their color; if there was no border, they were told to sort them according to their shape. Correct responses were scored as 1, incorrect answers as 0. The score for *shifting* corresponded to accuracy in Block 2 (i.e., ability to adapt to the new sorting rule). The score for *switching* corresponded to accuracy Block 3, in control items (i.e., unambiguous items: both the color and shape direct to the correct response) and test items (i.e., ambiguous items; the presence/absence of the stimulus’ border is decisive) separately.

### Analyses

We examined the impact of language experience on six EF tasks using a Bayesian multilevel modeling approach. Unlike frequentist methods, Bayesian methods provide more flexible modeling options by estimating full probability distributions for the variables of interest rather than relying solely on point estimates (Bürkner, 2018; Gelman et al., 2021). We chose Bayesian analysis for its ability to provide detailed uncertainty quantification and facilitate robust model comparisons through metrics such as leave-one-out cross-validation (LOO). These features make Bayesian methods particularly suited to studying the complex, multilevel nature of bilingualism and its impact on EF. The aims were to assess the model with the best fit to the data in each of the six EF tasks (RQ1), before investigating group differences between autistic and neurotypical children, and assessing the associations between varying bilingual experiences and EF task performance (RQ2). In case the models showed an interaction with *group*, we fitted the same model to each group’s data separately to examine the nature of this interaction.

#### Model Specifications.

Dependent variables were performance in six EF tasks: *SWAN* parent-reported (continuous) attention abilities (Task 1); accuracy (correct/incorrect) in the Simon task for congruent and incongruent conditions (Task 2); accuracy (correct/incorrect) of forward recall in the Frogs Matrices task (Task 3); accuracy (correct/incorrect) of backward recall in the Frogs Matrices task (Task 4); accuracy (correct/incorrect) in the shifting condition of the *DCCS* task (Task 5); accuracy (correct/incorrect) in the switching condition of the *DCCS* task (Task 6). The independent variables (bilingual predictors) were task-specific and operationalized based on the three theoretical accounts: To test the ICH (1), we examined *balance of cumulative use of different languages* as a predictor of performance on the inhibitory control task. To test the ACH (2), we examined *balance of current use within contexts, code-switching frequency, and their interaction*, as a predictor of performance on all six EF tasks. To test the EAA (3), we examined (3a) *balance of cumulative use of different languages*; (3b) *richness of exposure to and use of the second-best language*; (3c) and *proficiency in the second-best language*, as predictors of all six EF variables. Covariates included in all models were *chronological age*, *diagnostic group* (NT vs. autistic), *attention* (for all tasks except the attention task itself), and *Socioeconomic status*, measured through parental educational level. We also included the interaction of bilingual variables and *diagnostic group* to account for potential group-specific effects. This approach is grounded in prior evidence that EF skills differ in autistic and neurotypical populations (Demetriou et al., 2018; Wang et al., 2017). *Non-verbal IQ* and *attention* were included as covariates because they may serve as a fundamental skill underlying other executive function domains (Diamond, 2013). Furthermore, in the inhibitory control, STM, WM and shifting/switching tasks, *condition* was added to the two-way interaction between *diagnostic group* and the *bilingual predictor*, forming a three-way-interaction: In the inhibitory control task, condition distinguishes between congruent and incongruent items, in the STM and WM tasks between the “span”, i.e. the number of displayed frogs, and in the switching task between control and test items. All numeric fixed effects were scaled, to improve model convergence, facilitate interpretation of coefficients, and ensure comparability across predictors. Random intercepts were added for participants.

Bayesian models enable parameter estimation by incorporating both a hypothesized underlying generative process of the data, also known as the likelihood function, and prior knowledge about plausible parameter values. For Task 1, we used a normal likelihood function as the outcome variable was continuous, whereas for all other tasks, we employed a Bernoulli distribution to capture the binary nature of the corresponding outcome variables. We used weakly informative default priors. All models were fitted in *R* (R Core Team, 2019; version: 4.3.3), using the *brms* package (Bürkner, 2015). This package provides an interface to fit Bayesian generalized linear multilevel models using Stan as the backend for Markov Chain Monte Carlo sampling. For more details about the models, sample overviews, data, and analysis code, see OSF: https://osf.io/h8xav/?view_only=ebef23bb41bb4a16b2b7c084cefd3bb8.

#### Model Fitting.

We fitted all models using four chains with 10,000 iterations each. The adaptive delta parameter was set to 0.95 to minimize divergences. We evaluated convergence diagnostics, including *R̂* values, which should be close to 1.00, and effective sample sizes (Bulk ESS, the effective sample size in the central region of the posterior distribution, and Tail ESS, the effective sample size in the tails of the posterior distribution, should be above 1000; Bürkner, 2018; Gelman et al., 2021). For details on model evaluations see the Supplementary Materials.^3^

#### Model Comparisons.

For each task individually, we evaluated relative model fit by applying model comparisons. These comparisons allowed for a ranked evaluation of models to test hypotheses about the relative influence of bilingual predictors on EF outcomes. To determine for each EF task the model with the best fit, we compared several Bayesian multilevel models based on their relative predictive accuracy. Model comparison was conducted using leave-one-out cross-validation (LOO) and the expected log predictive density (ELPD). We evaluated model fit by considering two key indicators: (1) the highest ELPD value, reflecting better out-of-sample predictive performance and being directly related to the LOOIC; and (2) the effective number of parameters (p_loo), which captures the complexity of the model and its potential overfitting. We preferred one model over another if it demonstrated a better trade-off between predictive accuracy (high ELPD) and complexity (low p_loo). More detailed information on these model comparison characteristics is shown in the Supplementary Materials. While we do not draw strong conclusions about the theoretical hypotheses tested when model comparisons fail to provide compelling evidence for the superiority of one model over another, we reported the summary of the best-fitting model even when its fit differed only minimally from alternative models representing competing hypotheses.

## RESULTS

### Results From the Attention Task

#### Model Comparisons (RQ1).

To determine the best-fitting model for predicting attention, four models were compared: one based on the ACH (2), including *balance of current use within contexts* interacting with *code-switching frequency* as bilingual predictors, and three based on the executive attention model, including (3a) *balance of cumulative use*, (3b) *richness of exposure to and use of the second-best language*, and (3c) *proficiency in the second-best language* as bilingual predictors. Model comparisons revealed that Model 3c had the best relative fit to the data (see Supplementary Materials Table S2), based on the highest expected log predictive density (ELPD = −5797.7, *SE* = 60.6). The effective number of parameters for Model 3c (p_loo = 357.1, *SE* = 10.7) indicated a good balance between model complexity and predictive performance. Although the differences in ELPD between models were minimal, Model 3c had slight advantages over Model 3b (ELPD difference = −0.3, *SE* = 2.5) and Model 3a (ELPD difference = −0.8, *SE* = 2.3).

#### Model Outcomes (RQ2).

The outcome of the best model (3c; see Tables 2 and 3) revealed that autistic children had lower attention scores than the neurotypical group. The estimated effect was −1.10 (*SE* = 0.10), with a 95% CI of [−1.30, −0.91]. *L2 proficiency* was linked to higher attention ratings, with an estimated effect size of 0.23 (*SE* = 0.05) and a 95% CI of [0.13, 0.33]. The CI did not include zero, indicating a robust positive effect. *Socioeconomic status* had an estimated effect size of 0.00 (*SE* = 0.05), with a 95% CI range of [−0.09, 0.10], and *IQ* an effect size of 0.11 (*SE* = 0.05; 95% CI [0.02, 0.20]), suggesting that it was associated with better attention. Post-hoc analyses of the significant interaction between *L2 proficiency* and *diagnostic group* revealed that while L2 proficiency was associated with better attention in both autistic and neurotypical children, this was stronger in the autistic group.

**Table 2.** Result of Model 3c of attention.

	**Estimate (*SE*)**	**l-95% CI**	**u-95% CI**
Intercept	**0.01 (0.05)**	**−0.08**	**0.10**
**L2 proficiency**	**0.23 (0.05)**	**0.13**	**0.33**
**Diagnostic group**	**−1.10 (0.10)**	**−1.30**	**−0.91**
**SES**	**0.01 (0.05)**	**0.05**	**0.10**
**IQ**	**0.11 (0.05)**	**0.02**	**0.20**
L2 proficiency * diagnostic group	**0.16 (0.10)**	**0.05**	**0.41**

**Table 3.** Visualizations of the best fitting model of attention outcomes.

**PANEL 1.1**	**PANEL 1.2**
Conditional effects of *L2 proficiency* by *diagnostic group*	Posterior distribution of model parameters
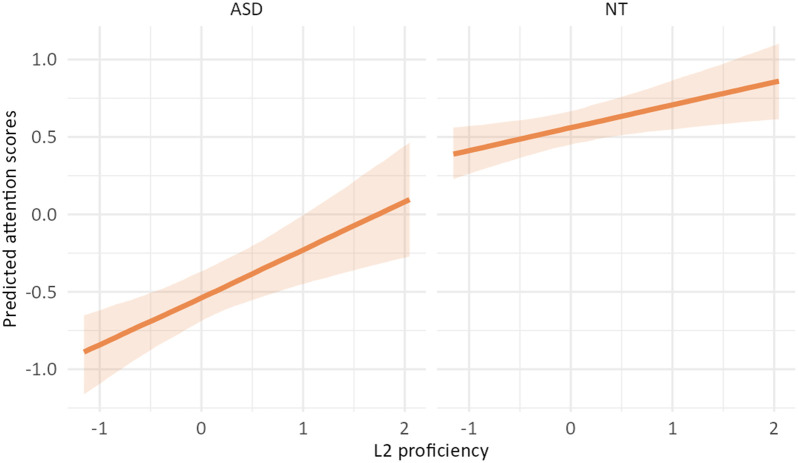	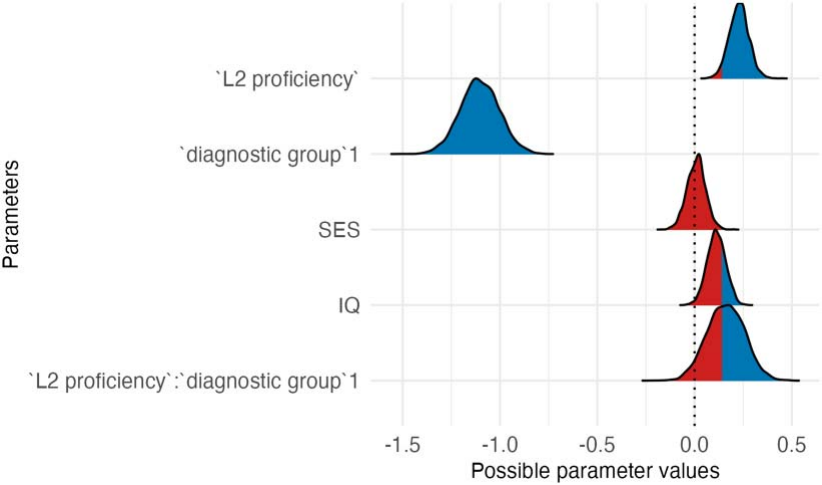
*Note*. This plot shows the relationship between L2 proficiency and predicted attention scores, separately for individuals with ASD and neurotypical participants. The solid orange line shows the estimated conditional effect of L2 proficiency on attention scores; the shaded ribbon indicates the 95% CI. A steeper slope suggests a stronger association between L2 proficiency and the outcome.	*Note*. This plot shows posterior distributions of model parameters. Color shading reflects the degree to which posterior mass lies away from zero: blue indicates parameter values further from zero, whereas red indicates values closer to zero. Parameters whose distributions are predominantly blue provide stronger evidence for an effect, while distributions containing substantial red shading indicate weaker or more uncertain evidence.

In summary, autistic children had lower attention scores than neurotypical children; higher L2 proficiency was linked to higher attention scores, particularly in the ASD group.

### Results From the Inhibitory Control Task

#### Model Comparisons (RQ1).

For predicting inhibitory control, we compared four models: one based on the ICH (Model 1), which was the same as Model 3a, including balance of cumulative use of different languages; one on the ACH (Model 2), including *balance of current use within contexts* interacting with *code-switching frequency*; and three based on the EAA, including *balance of cumulative use* (Model 3a), *richness of exposure to and use of the second-best language* (Model 3b), and *proficiency in the second-best language* (Model 3c) as bilingual predictors. Model comparisons, as shown in Table S3 in the Supplementary Materials, revealed that Model 3a had the best relative fit. For details, see the Supplementary Materials 3.

#### Model Outcomes (RQ2).

The results of the best model revealed that the groups did not differ on inhibitory control; see Tables 4 and 5. *Balance of cumulative use and exposure of different languages* was not associated with inhibitory control, as the CI included zero. Similarly, *congruency* (congruent vs. incongruent items), *Socioeconomic status, attention* and *IQ* were not linked to inhibitory control. Older children had better scores than younger children. Regarding the interaction between *balance of cumulative use of and exposure to different languages* and *congruency*, those with a higher balance of use and exposure seemed to show a smaller difference in performance between congruent and incongruent trials (i.e., better ability to inhibit the irrelevant information in incongruent trials).

**Table 4.** Result of Model 3a of inhibitory control.

	**Estimate (*SE*)**	**l-95% CI**	**u-95% CI**
**Intercept**	**3.03 (0.08)**	**2.88**	**3.20**
Balance of use/exposure	0.03 (0.08)	−0.12	0.19
Congruency (congruent vs. incongruent)	−0.12 (0.07)	−0.26	0.02
Diagnostic group (neurotypical vs. ASD)	0.03 (0.18)	−0.33	0.39
SES	0.00 (0.08)	−0.15	0.14
**Age**	**0.46 (0.08)**	**0.30**	**0.61**
Attention	0.13 (0.08)	−0.04	0.29
IQ	0.12 (0.07)	−0.03	0.26
Use/exp * congruency	**0.24 (0.07)**	**0.09**	**0.38**
Use/exp * diagnostic group	0.08 (0.16)	−0.23	0.39
Congruency * diagnostic group	−0.26 (0.14)	−0.54	0.03
Diagnostic group * age	0.08 (0.16)	−0.24	0.39
Use/exp * congruency * diagnostic group	0.00 (0.15)	−0.29	0.30

**Table 5.** Visualizations of the best fitting model (Model 1/3a) of inhibitory control outcomes.

**PANEL 2.1**	**PANEL 2.2**
Conditional effects of *balance of cumulative use* by *item type*	Posterior distribution of model parameters
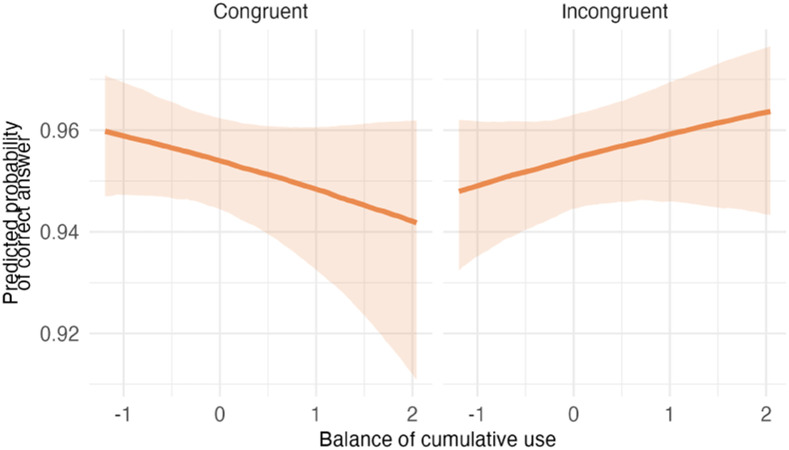	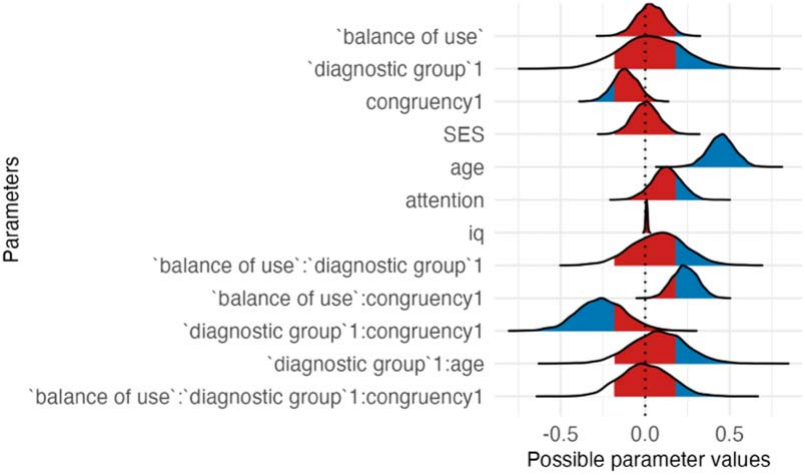

Although *balance of cumulative use and exposure* was not a significant predictor when congruent and incongruent trials were examined separately (congruent (95% CI [−0.25, 0.16]); incongruent items (95% CI [−0.07, 0.30]), its significant interaction with congruency (95% CI [0.09, 0.38]) indicates that children with higher balanced use showed a smaller interference effect. That is, they performed relatively better on incongruent trials compared to congruent trials, consistent with more efficient conflict resolution.

To sum up, autistic and neurotypical children did not differ in overall performance on the inhibitory control task. Although *balance of cumulative language use* was not associated with overall accuracy, a significant *balance* × *congruency* interaction indicated that greater balance was associated with a reduced congruency effect.

### Results From the Short-Term Memory Task

#### Model Comparisons (RQ1).

To predict STM, we compared four models: one based on the ACH (Model 2), including *balance of current use within contexts*, interacting with *code-switching frequency*, and three based on the executive attention model, including *balance of cumulative use of different languages* (Model 3a), *richness of exposure to and use of the second-best language* (Model 3b), and *proficiency in the second-best language* (Model 3c) as bilingual predictors. Model comparisons revealed that Model 3b had the best relative fit to the data; see Supplementary Materials Table S4.

#### Model Outcomes (RQ2).

The results of the best model revealed that NT children had better short-term memory scores than autistic children; see Tables 6 and 7. While *L2 richness* was not linked to short-term memory, post-hoc analyses of the interaction between *L2 richness* and *diagnostic group* showed no association between *L2 richness* and STM in NT children (95% CI [−0.25, 0.15]) but a positive association between *L2 richness* and STM in autistic children (95% CI [−0.03, 0.79]). Increasing *number of frogs* was associated with lower STM scores, suggesting that the accuracy dropped when the amount of information to be kept in short-term memory increased, particularly in ASD. Older *age*, higher *attention* abilities and higher *IQ* were positively linked to better STM.

**Table 6.** Result of Model 3b of short-term memory.

	**Estimate (*SE*)**	**l-95% CI**	**u-95% CI**
**Intercept**	**0.22 (0.09)**	**0.04**	**0.39**
L2 richness	0.10 (0.09)	−0.07	0.28
**Number frogs**	**−1.88 (0.08)**	**−2.03**	**−1.73**
**Diagnostic group (NT vs. ASD)**	**−0.46 (0.21)**	**−0.88**	**−0.04**
SES	0.04 (0.09)	−0.14	0.21
**Age**	**1.38 (0.10)**	**1.19**	**1.59**
**Attention**	**0.24 (0.10)**	**0.05**	**0.43**
**IQ**	**0.41 (0.09)**	**0.25**	**0.58**
L2 richness * number frogs	−0.02 (0.07)	−0.16	0.11
L2 richness * diagnostic group	**0.47 (0.17)**	**0.14**	**0.81**
Number frogs * diagnostic group	0.13 (0.14)	−0.15	0.40
Diagnostic group * age	0.03 (0.20)	−0.37	0.42
L2 richness * number frogs * diagnostic group	−0.10 (0.13)	−0.36	0.16

**Table 7.** Visualizations of the best fitting model (Model 3b) of short-term memory outcomes.

**PANEL 3.1**	**PANEL 3.2**
Conditional effects of *L2 proficiency* by *diagnostic group*	Posterior distribution of model parameters
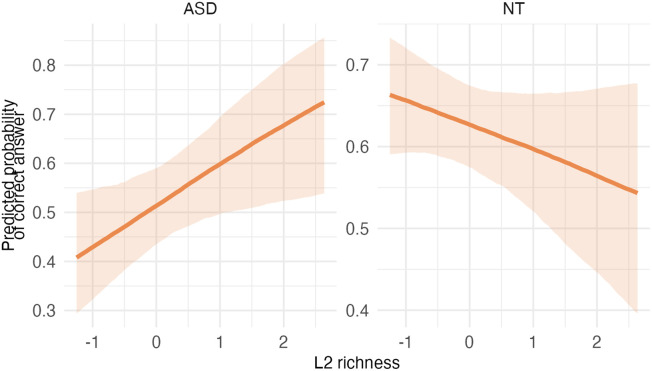	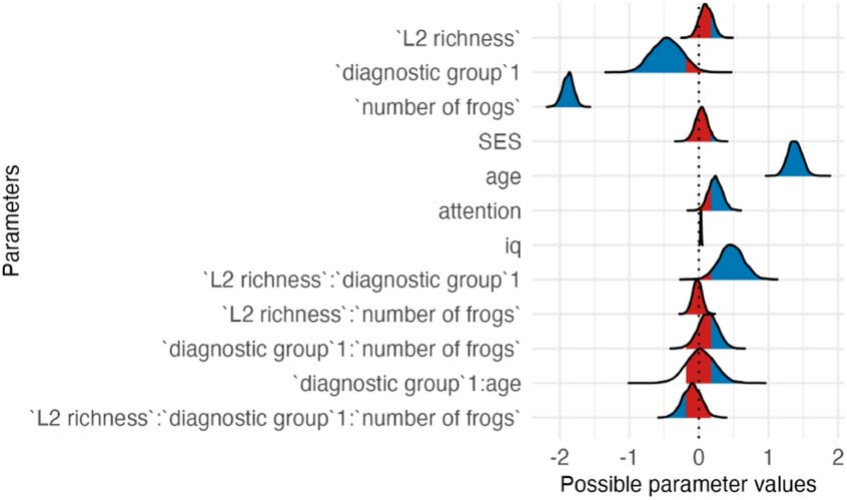

To sum up, autistic children had weaker STM than NT children, and higher L2 richness was associated with higher STM scores in autistic children. Furthermore, better attention, older age and higher IQ were also positively linked to better STM.

### Results From the Working Memory Task

#### Model Comparisons (RQ1).

To determine the best-fitting model for predicting WM, we compared four models: one based on the ACH (Model 2), including *balance of current use within contexts* interacting with *code-switching frequency*, and three based on the EAA, including *balance of cumulative use of different languages* (Model 3a), *richness of exposure to and use of the second-best language* (Model 3b), and *proficiency in the second-best language* (Model 3c) as bilingual predictors. Model comparisons revealed that Model 3c had the best relative fit; see Supplementary Materials Table S5.

#### Model Outcomes (RQ2).

The results of the best model revealed that autistic children performed weaker than NT children on WM (see Tables 8 and 9). *L2 proficiency* led to better WM. Increasing *number of frogs* was associated with lower WM scores, and *age* and higher IQ was positively linked to better WM. No interactions were found with *diagnostic group*.

**Table 8.** Result of Model 3c of working memory.

	**Estimate (*SE*)**	**l-95% CI**	**u-95% CI**
**Intercept**	**−0.79 (0.11)**	**−1.01**	**−0.58**
**L2 proficiency**	**0.28 (0.11)**	**0.07**	**0.49**
**Number frogs**	**−1.51 (0.07)**	**−1.65**	**−1.37**
**Diagnostic group (NT vs. ASD)**	**−0.90 (0.25)**	**−1.40**	**−0.42**
SES	−0.04 (0.10)	−0.24	0.16
**Age**	**1.44 (0.12)**	**1.21**	**1.68**
Attention	0.05 (0.11)	−0.16	0.27
**IQ**	**0.48 (0.10)**	**0.29**	**0.67**
L2 proficiency * number frogs	0.08 (0.06)	−0.04	0.21
L2 proficiency * diagnostic group	0.38 (0.21)	−0.02	0.79
Number frogs * diagnostic group	0.17 (0.13)	−0.09	0.43
Diagnostic group * age	−0.18 (0.24)	−0.65	0.29
L2 proficiency * number frogs * diagnostic group	0.18 (0.12)	−0.07	0.42

**Table 9.** Visualizations of the best fitting model (Model 3c) of working memory outcomes.

**PANEL 4.1**	**PANEL 4.2**
Conditional effects of *L2 proficiency* by *diagnostic group*	Posterior distribution of model parameters
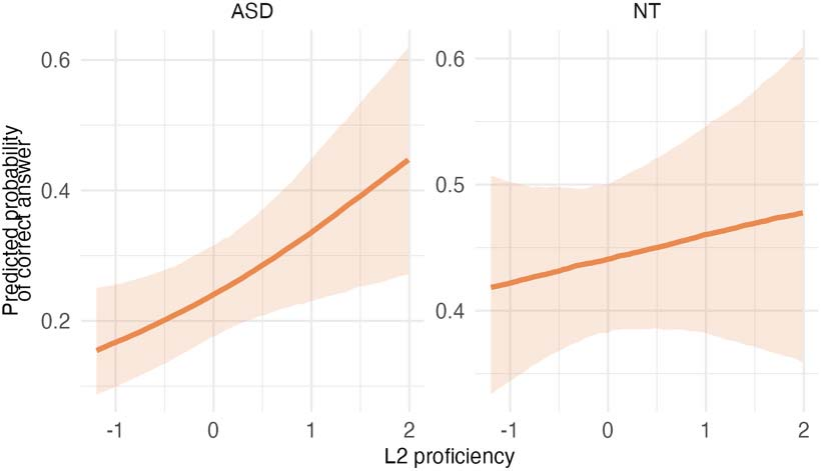	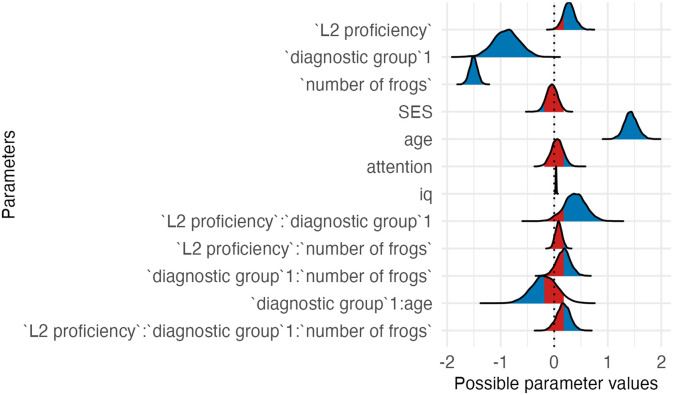

To sum up, autistic children had weaker WM than NT children; higher L2 proficiency was linked to higher WM scores.

### Results From the Shifting Task

#### Model Comparisons (RQ1).

To predict shifting, we compared four models: one based on the ACH (Model 2), including *balance of current use within contexts*, interacting with *code-switching frequency*, (Model 3a) *balance of cumulative use of different languages*, (Model 3b) *richness of exposure to and use of the second-best language*, and (Model 3c) *proficiency in the second-best language*. Model comparisons revealed that Model 3c had the best relative fit; see Table S6 in the Supplementary Materials.

#### Model Outcomes (RQ2).

The results of the best model (see Tables 10 and 11) revealed that *L2 proficiency* was not linked to shifting. Older *age* was positively associated with better shifting.

**Table 10.** Result of Model 3c of shifting.

	**Estimate (*SE*)**	**l-95% CI**	**u-95% CI**
**Intercept**	**3.03 (0.08)**	**2.87**	**3.19**
L2 proficiency	0.03 (0.09)	−0.13	0.20
Diagnostic group (NT vs. ASD)	0.04 (0.19)	−0.32	0.41
SES	0.00 (0.07)	−0.14	0.15
**Age**	**0.45 (0.09)**	**0.28**	**0.62**
Attention	0.13 (0.09)	−0.04	0.30
IQ	0.11 (0.07)	−0.03	0.25
L2 proficiency * diagnostic group	0.13 (0.16)	−0.19	0.45
Diagnostic group * age	0.05 (0.18)	−0.30	0.39

**Table 11.** Visualizations of the best fitting model (Model 3c) of shifting outcomes.

**PANEL 5.1**	**PANEL 5.2**
Conditional effects of *L2 proficiency* by *diagnostic group*	Posterior distribution of model parameters
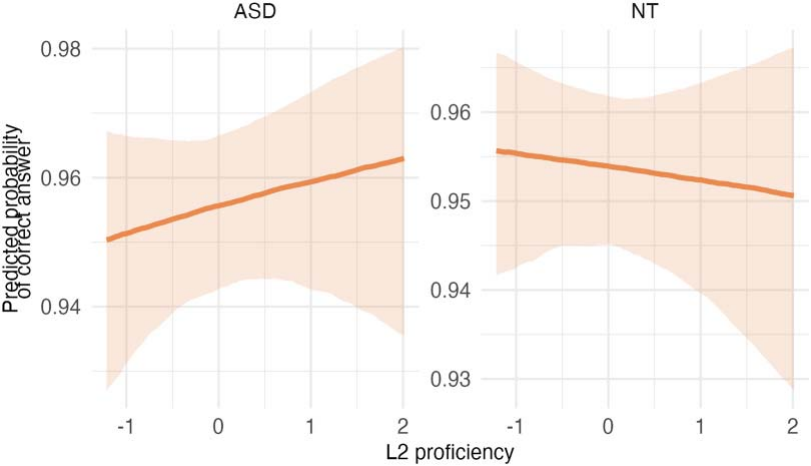	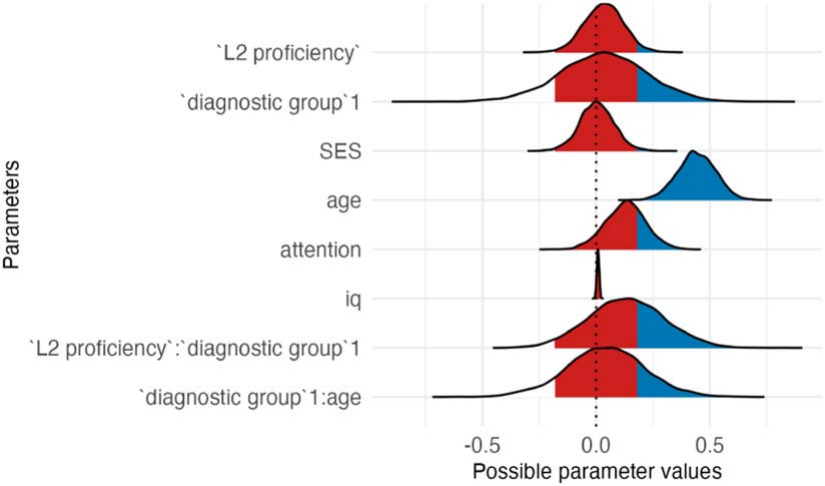

### Results From the Switching Task

#### Model Comparisons (RQ1).

For predicting switching, we compared four models: one based on the ACH (Model 2), including *balance of current use within contexts* interacting with *code-switching frequency*, and three based on the EAA, including *balance of cumulative use of different languages* (Model 3a), *richness of exposure to and use of the second-best language* (Model 3b), and *proficiency in the second-best language* (Model 3c) as bilingual predictors. Model comparisons, as shown in Table S7 in the Supplementary Materials, revealed that Model 3c had the best relative fit.

#### Model Outcomes (RQ2).

The results of Model 3c revealed that overall, the two groups did not differ in switching ability (see Tables 12 and 13). *L2 proficiency* was positively associated with switching performance, as was younger *age*. Although the simple slopes for control and test trials separately included zero, the significant interaction between *L2 proficiency* and item *type* (95% CI [−1.04, −0.26]) demonstrates that *L2 proficiency* differentially influenced performance across trial types. Children with higher *L2 proficiency* showed better accuracy on unambiguous control trials but lower accuracy on test trials, resulting in a larger performance difference between control and test trials (i.e., a larger switching cost).

**Table 12.** Result of Model 3c of switching.

	**Estimate (*SE*)**	**l-95% CI**	**u-95% CI**
**Intercept**	**1.29 (0.09)**	**1.11**	**1.47**
**L2 proficiency**	**0.19 (0.09)**	**0.01**	**0.36**
Diagnostic group (NT vs. ASD)	−0.16 (0.19)	−0.56	0.24
**Type (control vs. test)**	**−4.37 (0.15)**	**−4.69**	**−4.07**
SES	−0.06 (0.06)	−0.19	0.08
**Age**	**−0.26 (0.07)**	**−0.41**	**−0.10**
Attention	−0.03 (0.07)	−0.19	0.13
IQ	−0.13 (0.07)	−0.26	0.00
L2 proficiency * diagnostic group	−0.10 (0.18)	−0.44	0.25
L2 proficiency * type	**−0.75 (0.15)**	**−1.04**	**−0.26**
Diagnostic group * type	−0.00 (0.30)	−0.60	0.57
Diagnostic group * age	0.17 (0.16)	−0.13	0.49
L2 proficiency * diagnostic group * type	−0.19 (0.30)	−0.77	0.38

**Table 13.** Visualizations of the best fitting model (Model 3c) of switching outcomes.

**PANEL 6.1**	**PANEL 6.2**
Conditional effects of *L2 proficiency* by *condition*	Posterior distribution of model parameters
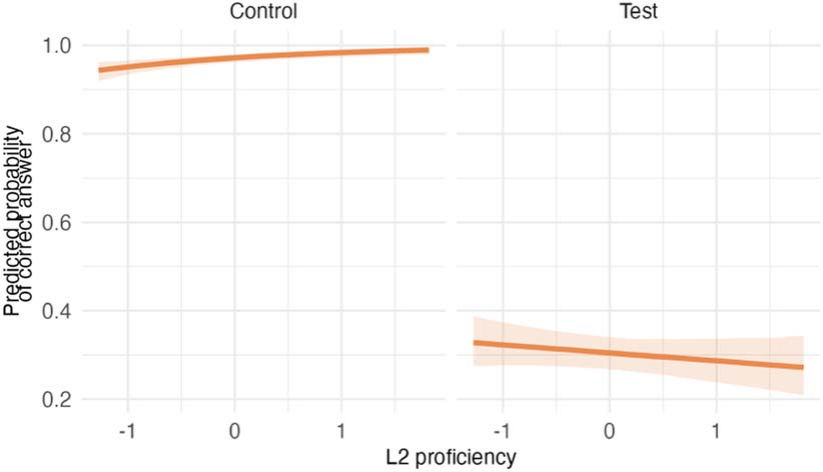	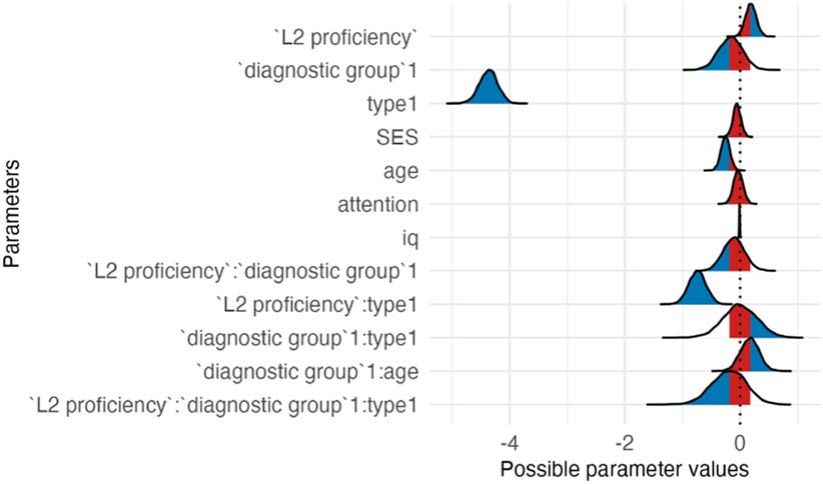

### Summary of the Results

A summary of all results is shown in Table 14.

**Table 14.** Summary of results.

	**Diagnostic group difference**	**Effect of language experience**
**Attention**	ASD < NT	Positive effect of L2 proficiency in NT and ASD, stronger in ASD
**Inhibitory control**	ASD = NT	Higher balance of use and exposure associated with a smaller interference effect
**Short-term memory**	ASD < NT	Positive effect of L2 richness in ASD
**Working memory**	ASD < NT	Positive effect of L2 proficiency in NT and ASD
**Shifting**	ASD = NT	No effect
**Switching**	ASD = NT	L2 proficiency was associated with higher accuracy on control trials but a larger switching cost in NT and ASD

## DISCUSSION

To test which theoretical framework best explains the relationship between bilingualism and EF in autistic and NT children, 168 autistic and 262 NT children completed a battery of six EF tasks. Their caregivers provided data on language experience across up to three languages. This information was used to operationalize key bilingualism-related factors based on the inhibitory control hypothesis (ICH), adaptive control hypothesis (ACH), and executive attention account (EAA).

### Discussion of Model Comparisons

Model comparisons revealed that while Model 3a showed the best relative fit for the inhibitory control task, Model 3b demonstrated the best fit for the STM task, and Model 3c provided the best fit for all other tasks.

### Impact of Bilingualism on Attention

Model comparisons showed that Model 3c, including *L2 proficiency* as the bilingual predictor, had the best fit to the data. Results showed that autistic children had lower attention scores than NT children, consistent with existing research (e.g., Berenguer et al., 2018; Narzisi et al., 2013). Furthermore, *L2 proficiency* led to higher attention scores, highlighting the impact of bilingualism on positive cognitive outcomes. This aligns with research suggesting that high L2 proficiency promotes attention development by requiring individuals to be attentive to language switches in their surroundings (Bialystok & Craik, 2022).

### Impact of Bilingualism on Inhibitory Control

Model 3a had the best relative fit, suggesting that *balance of cumulative use of different languages* may be a better predictor of inhibitory control than other aspects of bilingual experience. Although this model demonstrated the best fit to the data, the results indicated that *balance of cumulative use* was not associated with inhibitory control. A potential explanation for this finding is a ceiling effect in task performance, visible in Figure 2, Panel 2.1. Limited variability reduces sensitivity to and may have masked an influence of bilingual experience, including *balance of cumulative use*, on inhibitory control in this sample. This explanation is further supported by the absence of a difference between autistic and NT children, even though children with ASD generally demonstrate difficulties in inhibitory control (e.g., Kouklari et al., 2019). Although the best-fitting model included *balance of cumulative use*, the direct effect did not meet criteria for a main effect when examined by item type. However, the significant interaction between *balance of use* and *congruency* suggests that children with more balanced experience exhibited reduced interference costs. Given ceiling-level accuracy on congruent trials, the size of this interaction should be interpreted with caution.

### Impact of Bilingualism on Short-Term Memory

While *L2 richness* was not linked to short-term memory in NT children, it was positively associated with better STM in autistic children. Furthermore, ASD diagnosis was linked to lower STM outcomes, and greater attention abilities resulted in higher STM scores. These findings align with existing research indicating that STM abilities are often reduced in autistic children compared to neurotypical peers, consistent with broader cognitive challenges associated with autism (American Psychiatric Association, 2022). The positive link between STM and attention suggests that attentional control supports memory encoding and retrieval processes, as proposed in prior models of EF (e.g., Baddeley et al., 2020). The positive relationship between *L2 richness* and STM in autistic children highlights the potential benefits of bilingual experience for memory-related processes in this population. Receiving high quality input and having qualitatively rich activities in a second language may require and reinforce memory-related skills, such as retaining and manipulating information across two linguistic systems. This aligns with Bialystok’s (2017) hypothesis that the cognitive demands of bilingualism can enhance EF components, including STM, and this may especially hold for ASD. The lack of association between *L2 richness* and STM in neurotypical children likely reflected generally higher STM performance among neurotypical children.

### Impact of Bilingualism on Working Memory

Analyses of Model 3c, which had the best fit to the WM data, suggest that *L2 proficiency* contributed to enhanced WM performance. This finding supports the hypothesis that bilingual experience, particularly the development of proficiency, could strengthen EF like WM. As this task was more challenging than the STM task, the positive association between L2 proficiency and performance was evident in both groups. Consistent with previous work (Morales et al., 2013), this implies that the impact of bilingualism may be more evident in more challenging tasks. The finding that autistic children had lower outcomes than NT children aligns with previous research showing reduced WM capacity in autistic individuals compared to neurotypical peers (e.g., Kouklari et al., 2019).

### Impact of Bilingualism on Shifting

The evaluation of Model 3c, which had the best fit to the shifting data, indicated that autistic children performed equally well as neurotypical children. The absence of an association between *L2 proficiency* and shifting in both groups may indicate that their shifting abilities are already high, making them less sensitive to the additional cognitive demands of bilingualism in this domain, also demonstrated by close to ceiling performance.

### Impact of Bilingualism on Switching

The results of Model 3c suggested no differences for autistic and NT children on the switching task. It is important to note that only autistic children who had passed the shifting task were shown the switching task; thus, only children having no difficulties in shifting completed the more challenging switching tasks. While *L2 proficiency* was associated with overall better switching performance, the form of the effect was not uniform. The significant interaction between *L2 proficiency* and item *type* indicated that higher L2 proficiency enhanced accuracy on unambiguous control trials but slightly reduced accuracy on ambiguous test trials. One possible interpretation is that children with higher L2 proficiency relied more strongly on clear and consistent task cues, which supported performance when the correct response was obvious but made it more difficult to resolve trials in which stimulus dimensions conflicted and the border cue had to override competing information. Importantly, in our implementation of the border (mixed) block, control trials were unambiguous in that both stimulus dimensions directed toward the same response, placing relatively low demands on conflict resolution. Such trials primarily index rule maintenance and attention to task cues rather than switching under interference. Because accuracy on these trials was close to ceiling and showed limited variance, they contribute less information about individual differences in switching ability. As a result, the observed interaction may reflect differential effects of L2 proficiency on low-conflict versus high-conflict trials rather than a uniform influence on switching per se. Given this task structure and the limited variability in control performance, the magnitude of the interaction effect should therefore be interpreted with caution.

Taken together, the results suggest a consistent link between L2 proficiency and multiple aspects of executive functioning, particularly in autistic children. We interpreted L2 proficiency as a predictor of EF outcomes in line with existing theoretical models; however, a critical question arises: although it is often assumed that higher L2 proficiency enhances EF, the reverse relationship may also be plausible. In both prior research and the present study, it remains possible that stronger EF skills may facilitate greater success in acquiring a second language (Shokrkon & Nicoladis, 2022). As such, we refrain from making direct causal claims about the relationship between L2 proficiency and EF skills. Future longitudinal research will be essential to clarify the directionality of this relationship.

### Conclusion

This study explored the impact of hypothesis-driven bilingual predictors on EF in autistic and neurotypical children, drawing on multiple explanations of the reported “bilingualism advantage” in EF. Building on the nuanced ways in which bilingualism contributes to cognitive development, this work tested the predictions of three models, including the inhibitory control hypothesis (ICH; Green, 1998), the adaptive control hypothesis (ACH; Green & Abutalebi, 2013), and the executive attention account (Bialystok, 2017).

The first goal of this study was to build and compare models that would reflect these different hypotheses. Using Bayesian multilevel modeling, we assessed the models’ relative best fit to data collected on six different EF domains: attention, inhibitory control, short-term memory, working memory, shifting and switching. Findings revealed that for all EF tasks (except for inhibitory control and STM), models including *L2 proficiency* as the bilingual predictor presented the best fit to the data, highlighting the pivotal role of this variable on cognitive skills.

The second goal was to evaluate the associations between bilingualism (*L2 proficiency*, *balance of use/exposure*, L2 *richness*) and EF outcomes in both autistic and neurotypical children, exploring similarities and differences across these groups. Results revealed that *L2 proficiency* led to better attention, WM, and switching. Furthermore, *L2 richness* was associated with better STM. This underscores the relationship between *L2 proficiency* and EF. These findings suggest that autistic children, who generally experience EF difficulties, may benefit from growing up with or learning a second language, which could serve as a compensatory factor in their cognitive development.

This study was subject to several limitations. First, most participants spoke an Indo-European language, raising the question of whether findings would generalize to speakers of other language families, particularly those with typologically different linguistic structures. Future research could benefit from examining bilingualism and EF across a broader range of language groups. Additionally, our study primarily involved participants with average or above-average cognitive functioning, leaving open the question of how bilingualism might affect EF and other cognitive processes in populations with lower average cognitive scores, such as individuals with learning difficulties or developmental delays. It remains unclear whether the same patterns would emerge, or whether bilingualism might offer different cognitive outcomes in these groups. Furthermore, there are other contextual and individual differences (e.g., cultural attitudes toward bilingualism) that were not exhaustively captured in the present sample, but could moderate the relationship between bilingualism and cognitive functioning (Luk & Grundy, 2020). Specifically, while our sample was intentionally recruited across multiple countries (Canada, France, Germany, Spain, Switzerland, the United Kingdom and the United States) to enhance the ecological validity and generalizability of findings, the current study did not include a country-level breakdown of linguistic context or stratified analysis by site. This is primarily because the focus was on individual-level bilingual experiences. However, we acknowledge that national linguistic environments (e.g., societal multilingualism, education policies, minority language support) could influence language exposure patterns and EF outcomes. Preliminary analyses of the potential association between the country of residence and EF showed that no notable differences emerged (see Supplementary Materials 2).

Finally, we acknowledge that the SWAN is an informant-based measure developed primarily to assess attentional symptoms associated with ADHD, and differs methodologically from the performance-based EF tasks used in the present study (e.g., Simon, Frog Matrices, DCCS). That said, the SWAN has been shown to capture trait-like variations in attentional control across individuals with both low and high levels of attentional ability, not only those who meet clinical criteria for attention disorders (Swanson et al., 2012).

Our findings suggest that L2 proficiency emerges as a key bilingualism-related factor associated with EF development in both autistic and neurotypical children, highlighting the potential cognitive benefits of bilingual proficiency, in addition to its overarching social benefits.

## ACKNOWLEDGMENTS

We warmly thank all families who supported this research with their participation in our study and all institutions for their help in reaching out to participants. We also thank our collaborators who supported the data collection across Europe and North America.

## FUNDING INFORMATION

This work was supported by the Swiss National Science Foundation (SNSF) with a PRIMA grant awarded to Stephanie Durrleman (number: PR00P1_193104/1).

## AUTHOR CONTRIBUTIONS

F.B.: Conceptualization; Data curation; Formal analysis; Investigation; Methodology; Project administration; Supervision; Visualization; Writing – original draft. P.W.: Investigation; Methodology; Project administration; Supervision; Writing – review & editing. E.V.B.: Investigation; Writing – review & editing. E.S.: Conceptualization; Formal analysis; Writing – review & editing. I.M.E.: Conceptualization; Formal analysis; Writing – review & editing. S.D.: Conceptualization; Funding acquisition; Methodology; Project administration; Resources; Supervision, Writing – review & editing.

## DATA AND CODE AVAILABILITY STATEMENTS

The data and analytic code necessary to reproduce the analyses presented here are publicly accessible: https://osf.io/h8xav/?view_only=ebef23bb41bb4a16b2b7c084cefd3bb8.

The materials necessary to attempt to replicate the findings presented here are not publicly accessible; the analyses presented were not preregistered.

## COMPLIANCE WITH ETHICAL STANDARDS AND INFORMED CONSENT

This study was approved by the Swiss Association of Research Ethics Committees Swissethics (Project-ID 2022-00878), the Institutional Review Board of the University of Connecticut (US), the Research Ethics Board Office at McGill University in Montreal (Canada), the Psychology Research Ethics Committee of the University of Edinburgh (UK), the Institutional Review Board of Emerson College Boston (US), and the Research Ethics Committee of the Unió Catalana Hospitals Foundation (Spain).

## Notes

^1^ We did not include all bilingual predictors simultaneously in a single model because these variables reflect theoretically distinct, partially overlapping constructs. Entering them together would introduce multicollinearity and obscure the theoretical mechanisms each account proposes. Our goal was therefore not to identify the single “best” bilingual predictor, but to test the plausibility of distinct accounts. This theory-driven mapping between predictors and EF components avoids arbitrary model selection and ensures interpretability.^2^ As all EF tasks were minimally verbal, and the simple instructions were clearly understood by all participants, we decided not to control for proficiency in the language of testing. Furthermore, for the same reason, we also did not include a measure of verbal IQ in our study protocol. In addition, this choice aligns with recommendations to prioritise non-verbal measures in ASD to avoid linguistic confounds (Silleresi, 2023). Empirical work shows that verbal IQ tests may underestimate cognitive ability in autistic children, whereas non-verbal reasoning tasks such as the Raven’s provides a more valid estimate of general ability (e.g., Nader et al., 2016).^3^ The sample size for this study was determined by the available data from a larger research project investigating the impact of bilingualism on cognitive and communicative skills in children with and without autism. For this study, data from 168 autistic and 262 neurotypical children were available.While we did not conduct a priori power analyses, which are less applicable in Bayesian modeling, we assessed model adequacy using convergence diagnostics. All models showed excellent convergence with R-hat = 1.00 for all parameters and effective sample sizes well above recommended thresholds (most Bulk and Tail ESS > 10,000, some > 50,000), as presented in the Supplementary Materials. These results indicate that our models were well-calibrated and that the available data were sufficient for robust posterior estimation. Additionally, we used posterior predictive checks and leave-one-out cross-validation to ensure model fit and stability. These diagnostics provide strong evidence that the sample size was sufficient to support the complexity of the Bayesian multilevel models employed.

## Supplementary Material


