# Supplementary material for: Associations Between Second-Language Proficiency and Executive Functions in Autistic and Neurotypical Children
Source: Open Mind (Camb). 2026 Apr 17;10:613–40. doi: 10.1162/OPMI.a.349 (PMC13233095; doi:10.1162/OPMI.a.349)
Supplement: Supplementary file 1 [file opmi-10-613-s001.pdf]

Supplementary Material 1 Participant characteristics

Table A1.1

Overview of participants' biographic information per task

	N		**Age**<br>In years *M* (*SD*)<br>range		**Gender**<br>*n* Diverse<br>*n* Female<br>*n* Male		**Parental educational level**<br>*M* (*SD*)<br>range		**Attention**<br>(SWAN z-score)<br>*M* (*SD*)<br>range		**Non verbal IQ**<br>(Raven's 2)<br>M (*SD*) range		**Receptive vocabulary**<br>(PPVT-4)<br>M (*SD*) range	
	**ASD**	**NT**	**ASD**	**NT**	**ASD**	**NT**	**ASD**	**NT**	**ASD**	**NT**	**ASD**	**NT**	**ASD**	**NT**
**Task 1**<br>**Attention**<br>(SWAN questionnaire)	168	262	8;3 (2;3)<br>3;1 – 12;0	7;8 (2;5)<br>3;2 – 11;11	1<br>36<br>131	1<br>133<br>128	4.2 (1.1)<br>1 – 5	4.6 (0.7)<br>2 – 5	-0.6 (1.1)<br>-2.9 – 2.7	0.6 (0.8)<br>-1.0 – 3.0	94.6 (14.8)<br>66 – 133	99.3 (13.1)<br>66 – 133	-1.0 (1.8)<br>-4.0 – 4.0	0.4 (1.2)<br>-4.0 – 3.3
**Task 2**<br>**Inhibitory control**<br>(Simon task)	124	236	8;8 (2;2)<br>3;1 – 11;11	7;9 (2;5)<br>3;2 – 11;11	1<br>23<br>10	1<br>118<br>117	4.0 (1.1)<br>1 – 5	4.6 (0.7)<br>2 – 5	-0.5 (1.0)<br>-2.9 – 2.4	0.6 (0.8)<br>-0.9 – 3.0	94.6 (14.4)<br>66.0 – 133	99.2 (13.2)<br>66.0 – 133	-0.9 (1.8)<br>-4.0 – 3.3	0.4 (1.2)<br>-4.0 – 3.1
**Task 3**<br>**Visual STM**<br>(FMT, forward recall)	125	236	8;8 (2;2)<br>3;1 – 12;0	7;10 (2;4)<br>3;2 – 11;11	1<br>20<br>104	1<br>118<br>117	4.0 (1.2)<br>1 – 5	4.6 (0.7)<br>2 – 5	-0.5 (1.0)<br>-2.9 – 2.4	0.6 (0.8)<br>-0.9 – 3.0	95.3 (14.4)<br>66 – 133	99.2 (13.0)<br>66 – 133	-0.9 (1.8)<br>-4.0 – 4.00	0.4 (1.2)<br>-4.0 – 3.3
**Task 4**<br>**Visual WM**<br>(FMT, backward recall)	125	236	8;8 (2;2)<br>3;1 – 12;0	7;10 (2;4)<br>3;2 – 11;11	1<br>20<br>104	1<br>118<br>117	4.0 (1.2)<br>1 – 5	4.6 (0.7)<br>2 – 5	-0.5 (1.0)<br>-2.9 – 2.4	0.6 (0.8)<br>-0.9 – 3.0	95.3 (14.4)<br>66 – 133	99.2 (13.0)<br>66 – 133	-0.9 (1.8)<br>-4.0 – 4.00	0.4 (1.2)<br>-4.0 – 3.3
**Task 5**<br>**Shifting**<br>(DCCS, block 1 & 2)	137	246	7;9 (2;5)<br>3;2 – 11;11	8;7 (2;3)<br>3;1 – 12;0	1<br>24<br>112	1<br>123<br>122	4.0 (1.2)<br>1 – 5	4.6 (0.7)<br>2 – 5	-0.5 (1.1)<br>-2.9 – 2.4	0.6 (0.8)<br>-0.9 – 3.0	95.0 (14.7)<br>66 – 133	99.1 (13.2)<br>66 – 133	-0.9 (1.8)<br>-4.0 – 3.3	0.5 (1.2)<br>-4.0 – 4.0
**Task 6**<br>**Switching**<br>(DCCS, block 3)	85	193	9;3 (1;12)<br>4;4 – 11;11	8;2 (2;3)<br>3;10 – 11;11	1<br>17<br>67	1<br>103<br>89	4.1 (1.1)<br>1 – 5	4.6 (0.7)<br>2 – 5	-0.4 (1.1)<br>-2.8 – 2.4	0.6 (0.8)<br>-0.9 – 3.0	96.5 (14.1)<br>71 – 133	101 (12.8)<br>70 – 130	-0.4 (1.5)<br>-4.0 – 3.3	0.5 (1.1)<br>-4.0 – 3.1

Note. SD = Standard deviation; SWAN = Strengths and Weaknesses of Attention-Deficit/Hyperactivity Disorder Symptoms and Normal Behavior Scale (Swanson et al., 2012); FMT = Frogs Matrices Task (Morales et al., 2013); DCCS = Dimensional Change Card Sorting Task (Zelazo et al., 1996); Parental educational level: 1 (elementary school) and 5 (university degree); Standardized IQ scores are reported for the Raven's-2 (Raven et al., 2018) assessing non-verbal IQ; Z-scores are reported for the PPVT-4 = Peabody Picture Vocabulary Test. 4th Edition (Dunn and Dunn, 2007), assessing receptive vocabulary in the testing language. Attention is listed as a control variable for all tasks (except "Task 1 Attention") as this factor is included as a covariate in the statistical analyses for all other tasks, due to its impact on other EFs.

Table A1.2

Overview of participants’ language experiences

Bilingual dimension	**ASD**	**NT**	**Visualization**
**(1) Balance of cumulative use of different languages across contexts**	0.51 (0.49)<br>0.00 – 1.58	0.55 (0.50)<br>0.00 – 1.58	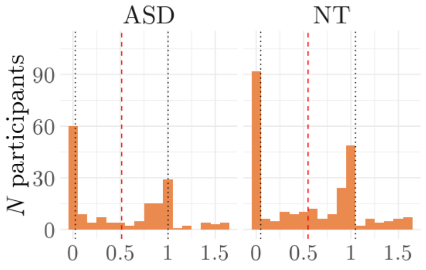
**(2) Balance of current use of different language within contexts**	0.45 (0.41)<br>0 – 1.58	0.43 (0.40)<br>0.00 – 1.45	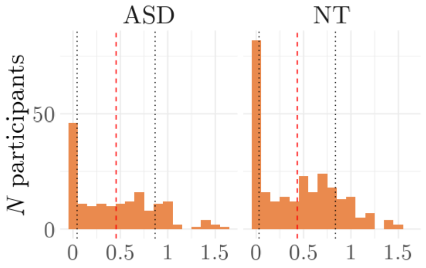
**(3) Code-switching frequency**	0.15 (0.28)<br>0.00 – 1.00	0.16 (0.28)<br>0.00 – 1.00	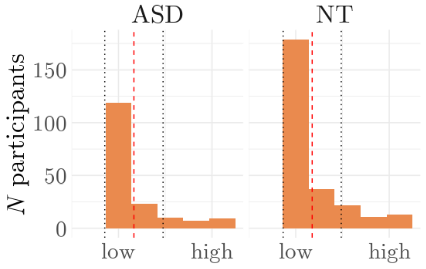
**(4) Richness of exposure to and use of the L2**	0.30 (0.23)<br>0.00 – 0.91	0.29 (0.24)<br>0.00 – 0.93	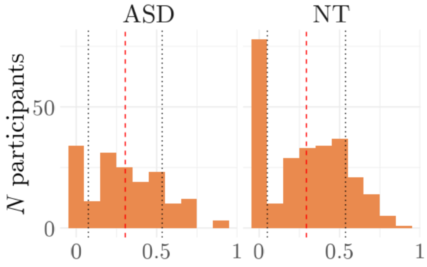
**(5) L2 proficiency**	0.33 (0.29)<br>0.00 – 1.00	0.38 (0.32)<br>0.00 – 1.00	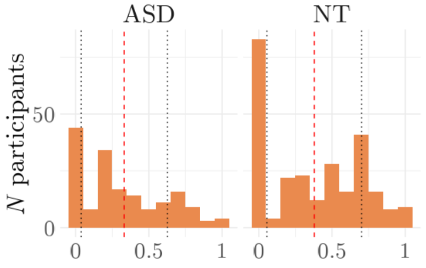

Note. ASD = autistic children, NT = neurotypical children; data reported as *M* (*SD*), range; red dashed line = mean value; black dashed lines = +/- 1 SD; L2 = second-best language.

Associations between second-language proficiency and executive functions in autistic and neurotypical children – SUPPLEMENTARY MATERIAL

Table A1.3

Overview of participants' bilingual characteristics

	NT children (N=262)	Autistic children (N=168)	All children (N=430)
**Language status**			
Monolingual	75 (28.6%)	34 (20.2%)	109 (25.3%)
Bilingual	126 (48.1%)	87 (51.8%)	213 (49.5%)
Trilingual	61 (23.3%)	47 (28.0%)	108 (25.2%)
**Age of First Exposure to L1 (months)**			
Mean (SD)	4.59 (14.2)	7.77 (17.0)	5.82 (15.4)
Median [Min, Max]	0 [0, 90]	0 [0, 95]	0 [0, 95]
Missing information N (%)	3 (1.1%)	4 (2.4%)	7 (1.6%)
**Age of First Exposure to L2 (months)**			
Mean (SD)	10.9 (21.3)	16.0 (28.0)	13.0 (24.4)
Median [Min, Max]	0 [0, 97]	0 [0, 130]	0 [0, 130]
**Age of First Exposure to L3 (months)**			
Mean (SD)	21.8 (32.4)	21.1 (28.6)	21.5 (30.7)
Median [Min, Max]	1 [0, 126]	6 [0, 104]	5 [0, 126]
**Place of First Exposure to L1**			
Home	216 (82.4%)	130 (77.4%)	346 (80.5%)
School	34 (13.0%)	26 (15.5%)	60 (14.0%)
Other	12 (4.6%)	12 (7.1%)	24 (5.6%)
**Place of First Exposure to L2**			
Home	113 (43.1%)	91 (54.2%)	204 (47.4%)
School	54 (20.6%)	33 (19.6%)	87 (20.2%)
Other	20 (7.6%)	10 (6.0%)	30 (7.0%)
**Place of First Exposure to L3**			
Home	32 (12.2%)	24 (14.3%)	56 (13.0%)
School	23 (8.8%)	18 (10.7%)	41 (9.5%)
Other	6 (2.3%)	5 (3.0%)	11 (2.6%)
**First Exposure type**			
Both languages at home	99 (53.2%)	77 (57.5%)	176 (54.8%)
Mixed exposure (home/school)	88 (46.8%)	57 (42.5%)	145 (45.2%)
**Bilingual status**			
Simultaneous bilingual (≤ 36 months)	172 (91.9%)	120 (89.6%)	292 (90.9%)
Sequential bilingual (> 36 months)	12 (6.4%)	10 (7.5%)	22 (6.9%)
Missing information N (%)	3 (1.6%)	4 (2.6%)	7 (2.2%)
**Testing language corresponded to…**			
L1 (Most proficient language)	220 (84.0%)	146 (86.9%)	366 (85.1%)
L2 (Second-best language)	37 (14.1%)	20 (11.9%)	57 (13.3%)
L3 (Third language)	5 (1.9%)	2 (1.2%)	7 (1.6%)

Figure A1.4.

Overview of the participants' diversity of languages

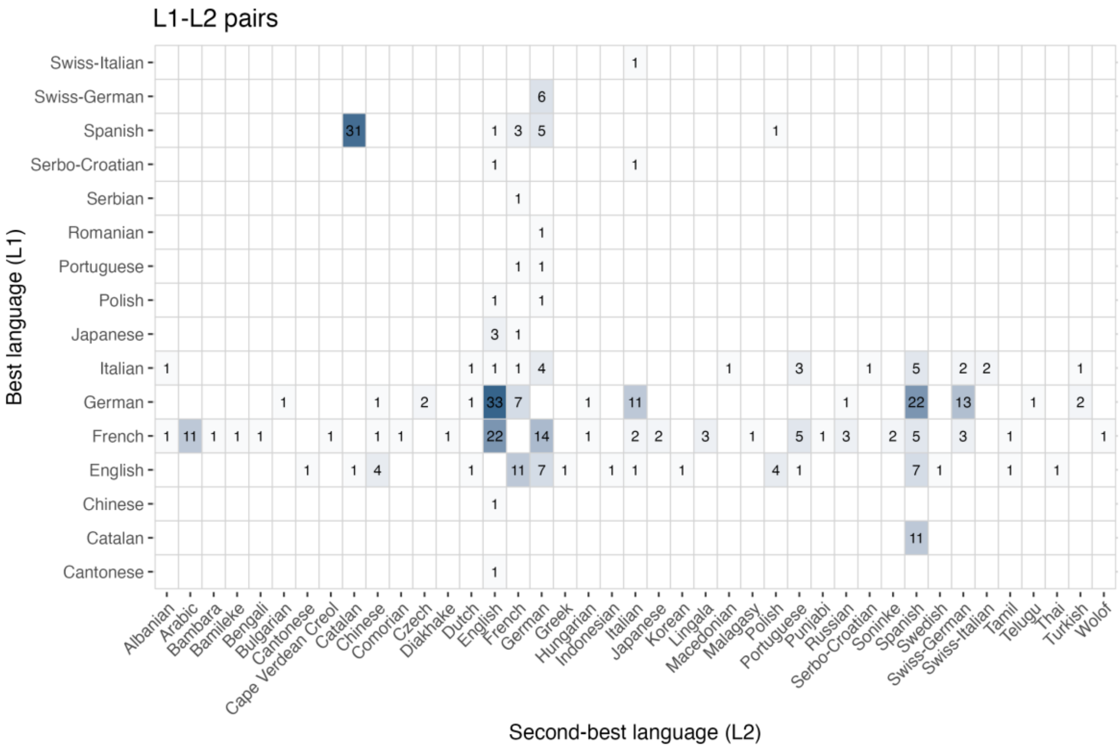

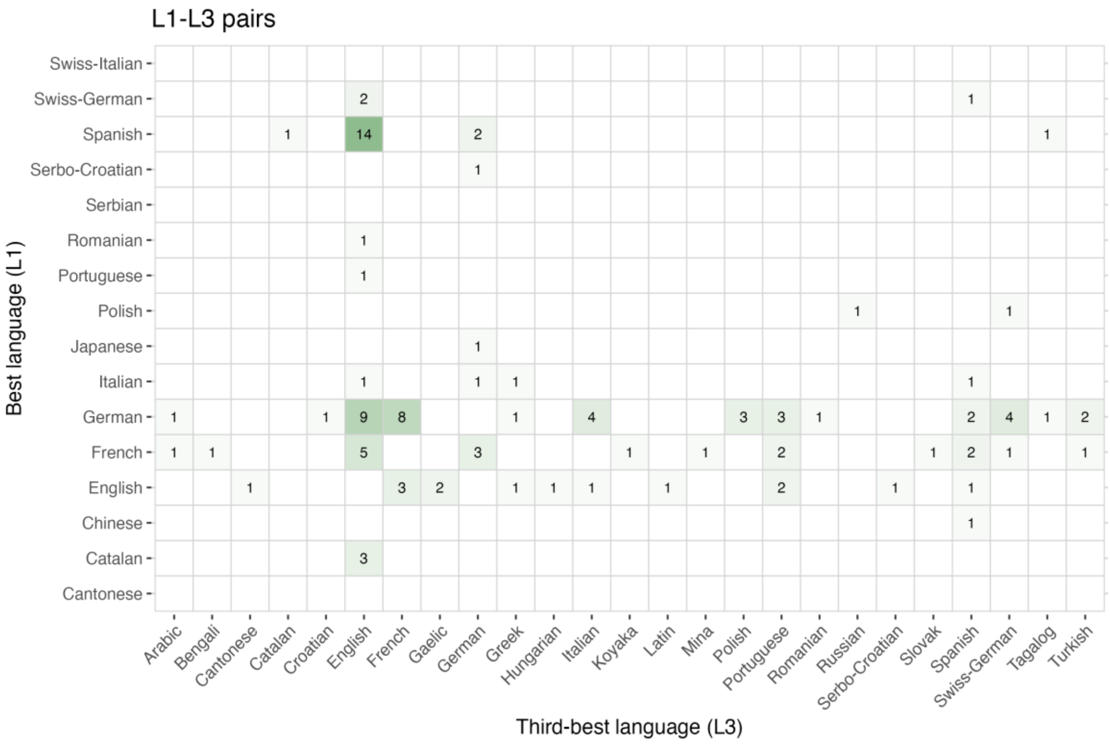

Supplementary Material 2 Task 1 (Attention)

Table A2.1
Model comparisons for Task 1

	elpd_diff	se_diff	elpd_loo	se_elpd_loo	p_loo	se_p_loo	looic	se_looic
**EA-proficiency-model**	0.0	0.0	-4780.4	55.3	296.7	9.7	9560.7	110.6
**EA-richness-model**	-0.7	1.5	-4781.1	55.3	297.7	9.7	9562.1	110.5
**EA-quantity-model**	-0.9	1.6	-4781.3	55.4	297.9	9.8	9562.5	110.9
**ACH-model**	-1.7	2.0	-4782.8	55.6	299.4	9.9	9564.1	111.2

Note. elpd_diff: difference in expected log predictive density (ELPD) between each model and the best-performing model (higher value indicates better predictive performance); se_diff: standard error of the ELPD difference, reflecting uncertainty in the comparison; elpd_loo: expected log predictive density for each model, estimated using LOO; e_elpd_loo: standard error of the ELPD estimate, indicating the uncertainty in model predictive performance; p_loo: effective number of parameters in each model, reflecting model complexity; se_p_loo: standard error of the effective number of parameters; looic: LOO; se_looic: standard error of the LOOIC estimate.

Posterior predictive checks

Posterior predictive checks were conducted for the model with the best fit (3c) to evaluate whether it adequately modeled the observed data; see Figure A2.1. Visual inspection of the posterior predictive distributions showed alignment with the observed data, indicating that the model effectively captured key patterns in the data. The checks confirmed that the Model (3c) could generate plausible values consistent with the observed outcomes.

Figure A2.1
Posterior predictive check for the best model of attention

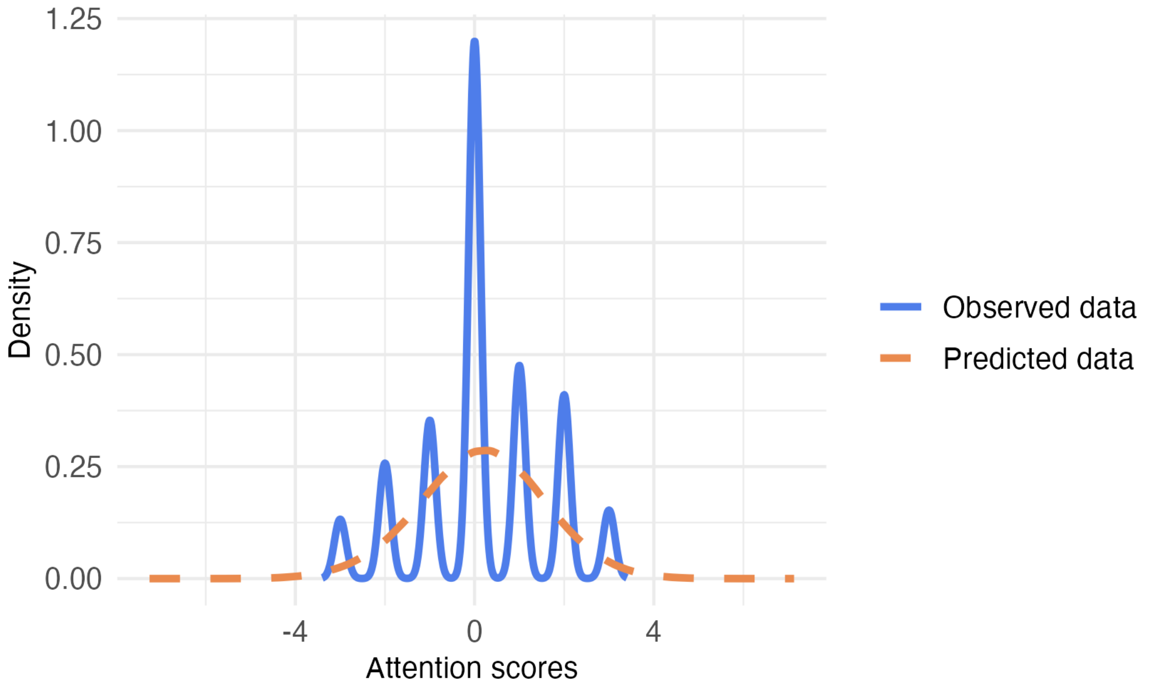

Note. This plot shows the comparison between the distribution of observed data distribution (blue) with the predicted distribution (orange). A close alignment between the two distributions suggests that the model is successfully capturing key characteristics of the observed data. Big discrepancies, such as deviations in peak location, spread, or skewness, may indicate model misfit, suggesting that some features of the data are not fully captured by the current specification.

Table A2.2
Result of Model (3c) on attention

	Estimate (SE)	l-95% CI	u-95% CI	Rhat	Bulk ESS	Tail ESS
Intercept	0.01 (0.05)	-0.08	0.10	1.00	5780	9393
**L2 proficiency**	**0.23 (0.05)**	**0.13**	**0.33**	**1.00**	**5018**	**9259**
**diagnostic group**	**-1.10 (0.10)**	**-1.30**	**-0.91**	**1.00**	**5094**	**8690**
SES	0.01 (0.05)	0.05	0.10	1.00	5257	8931
IQ	0.11 (0.05)	0.02	0.20	1.00	5535	9314
**L2 proficiency * diagnostic group**	**0.16 (0.10)**	**-0.03**	**0.35**	**1.00**	**5233**	**9402**

Effect of country of residence

To assess whether national context influenced EF outcomes, we reran the best-fitting model for attention with country of residence included as a fixed effect (using sum coding). Results showed that most country coefficients had 95% credible intervals overlapping with zero, indicating no strong evidence for differences in EF outcomes by country. One exception was Switzerland, which showed a modest positive association (Estimate = 0.26, 95% CI [0.08, 0.43]). However, the overall pattern suggests that individual bilingual experience, rather than country-level context, primarily accounted for EF variation in this sample.

Supplementary Material 3 Task 2 (Inhibitory control)

Table A3

Model comparisons for Task 2

	elpd_diff	se_diff	elpd_loo	se_elpd_loo	p_loo	se_p_loo	looic	se_looic
**EA-quantity-model**	0.0	0.0	-3540.0	77.8	213.6	6.9	7079.9	155.7
**EA-proficiency-model**	0.0	2.9	-3540.0	77.8	213.3	6.9	7080.0	155.6
**ACH-model**	-0.1	4.7	-3540.1	78.1	221.8	7.2	7080.2	156.3
**ICH-model**	-0.1	1.3	-3540.1	77.8	213.9	6.9	7080.2	155.5
**EA-richness-model**	-0.6	2.8	-3540.6	77.8	212.2	6.9	7081.1	155.6

Note. elpd_diff: difference in expected log predictive density (ELPD) between each model and the best-performing model (higher value indicates better predictive performance); se_diff: standard error of the ELPD difference, reflecting uncertainty in the comparison; elpd_loo: expected log predictive density for each model, estimated using LOO; e_elpd_loo: standard error of the ELPD estimate, indicating the uncertainty in model predictive performance; p_loo: effective number of parameters in each model, reflecting model complexity; se_p_loo: standard error of the effective number of parameters; looic: LOO; se_looic: standard error of the LOOIC estimate.

Posterior predictive checks

Posterior predictive checks were conducted for Model (3a) to show that the model adequately replicated the observed data; see Figure A3.1.

Figure A3.1

Posterior predictive check for the best model of inhibitory control

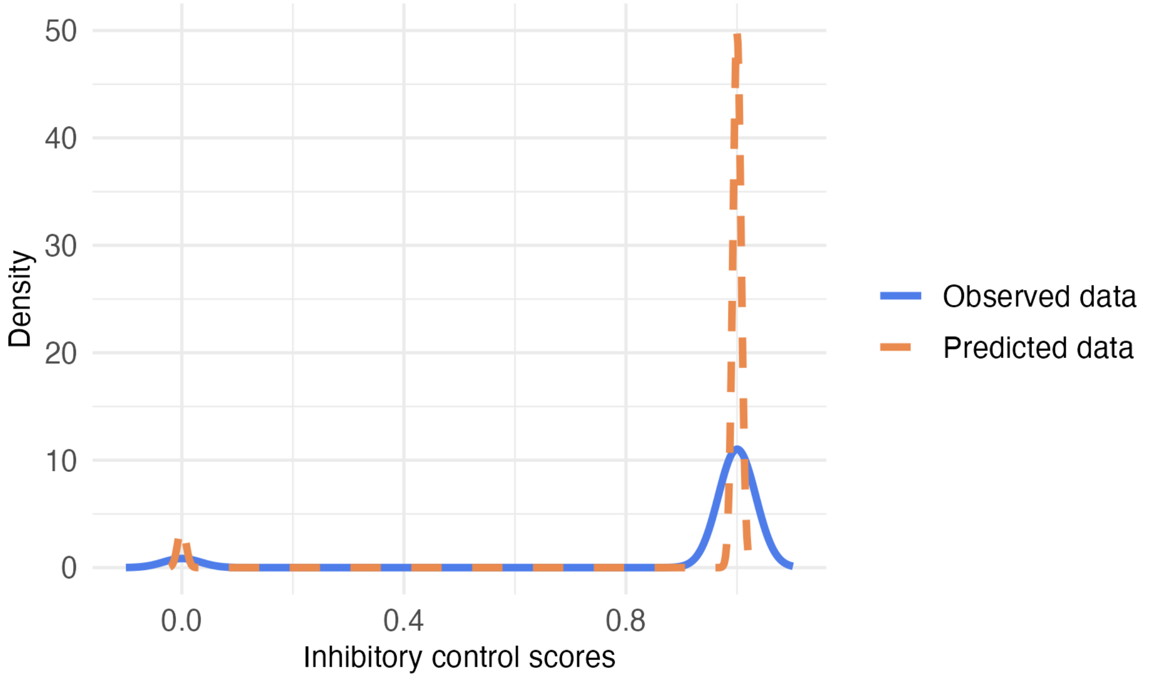

Table A3.2

Result of Model (1)/(3a) on inhibitory control

	Estimate (SE)	l-95% CI	u-95% CI	Rhat	Bulk ESS	Tail ESS
**Intercept**	**3.03 (0.08)**	**2.88**	**3.20**	**1**	**1949**	**2902**
balance of use/exposure	0.03 (0.08)	-0.12	0.19	1	1751	2206
congruency (congruent vs incongruent)	-0.12 (0.07)	-0.26	0.02	1	7545	2873
diagnostic group (NT vs ASD)	0.03 (0.18)	-0.33	0.39	1	1473	2397
SES	0.00 (0.08)	-0.15	0.14	1	1671	2474
**age**	**0.46 (0.08)**	**0.30**	**0.61**	**1**	**1756**	**2537**
attention	0.13 (0.08)	-0.04	0.29	1	1640	2342
IQ	0.12 (0.07)	-0.03	0.26	1	1730	2344
**use/exp * congruency**	**0.24 (0.07)**	**0.09**	**0.38**	**1**	**8237**	**2893**
use/exp * diagnostic group	0.08 (0.16)	-0.23	0.39	1	1740	2520
congruency * diagnostic group	-0.26 (0.14)	-0.54	0.03	1	7012	2370
diagnostic group * age	0.08 (0.16)	-0.24	0.39	1	1927	2436
use/exp * congruency * diagnostic group	0.00 (0.15)	-0.29	0.30	1	7834	2985

Supplementary Material 4 Task 3 (Short-term memory)

Table A4.1
Model comparisons for Task 3

	elpd_diff	se_diff	elpd_loo	se_elpd_loo	p_loo	se_p_loo	looic	se_looic
**EA-richness-model**	0.0	0.0	-1353.8	33.0	194.1	5.3	2707.7	66.8
**EA-proficiency-model**	-0.7	1.5	-1354.3	33.1	194.4	5.4	2709.1	66.2
**EA-quantity-model**	-1.4	2.1	-1355.3	32.8	196.7	5.4	2710.5	65.7
**ACH-model**	-5.0	3.3	-1358.8	33.2	206.5	5.8	2717.6	66.4

Note. elpd_diff: difference in expected log predictive density (ELPD) between each model and the best-performing model (higher value indicates better predictive performance); se_diff: standard error of the ELPD difference, reflecting uncertainty in the comparison; elpd_loo: expected log predictive density for each model, estimated using LOO; e_elpd_loo: standard error of the ELPD estimate, indicating the uncertainty in model predictive performance; p_loo: effective number of parameters in each model, reflecting model complexity; se_p_loo: standard error of the effective number of parameters; looic: LOO; se_looic: standard error of the LOOIC estimate.

Posterior predictive checks

Posterior predictive checks indicated that Model (3b) reliably captured the observed data; see Figure A4.1.

Figure A4.1
Posterior predictive check for the best model of STM

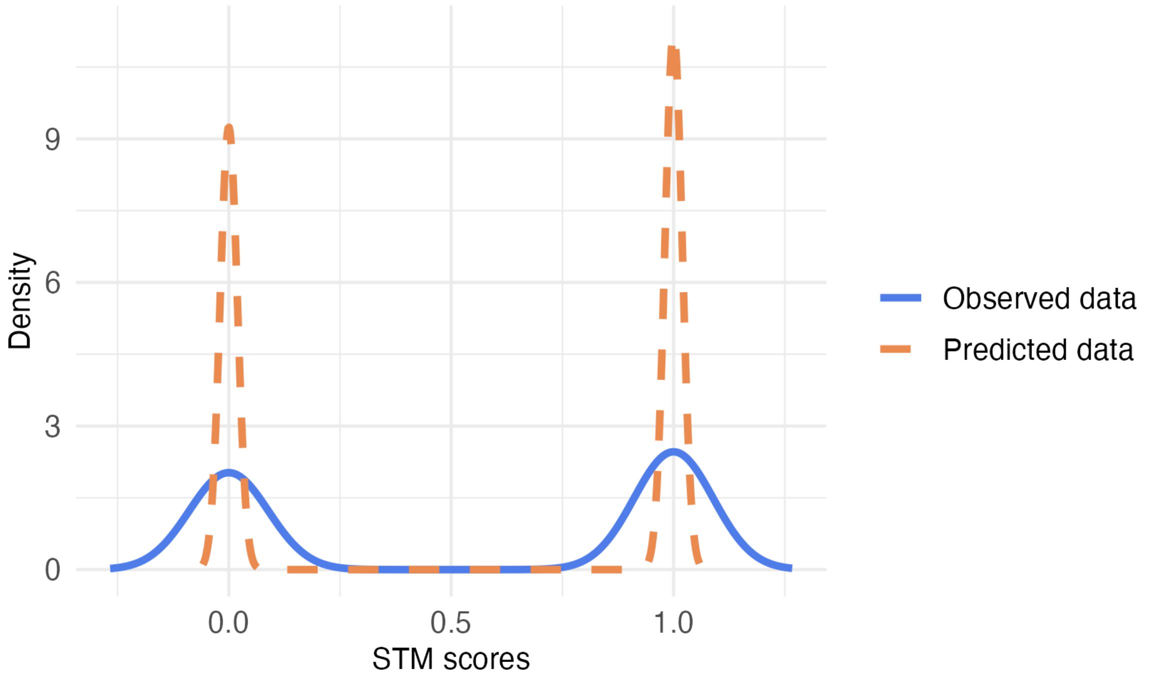

Table A4.2.

Result of model (3b) of short-term memory

	Estimate (SE)	l-95% CI	u-95% CI	Rhat	Bulk ESS	Tail ESS
**Intercept**	**0.22 (0.09)**	**0.04**	**0.39**	**1**	**18969**	**17275**
L2 richness	0.10 (0.09)	-0.07	0.28	1	18018	16523
**number frogs**	**-1.88 (0.08)**	**-2.03**	**-1.73**	**1**	**28595**	**16196**
**diagnostic group (NT vs ASD)**	**-0.46 (0.21)**	**-0.88**	**-0.04**	**1**	**17068**	**16052**
SES	0.04 (0.09)	-0.14	0.21	1	17938	16532
**age**	**1.38 (0.10)**	**1.19**	**1.59**	**1**	**18321**	**16875**
**attention**	**0.24 (0.10)**	**0.05**	**0.43**	**1**	**18297**	**17105**
**IQ**	**0.41 (0.09)**	**0.25**	**0.58**	**1**	**19312**	**16275**
L2 richness * number frogs	-0.02 (0.07)	-0.16	0.11	1	33961	16111
**L2 richness * diagnostic group**	**0.47 (0.17)**	**0.14**	**0.81**	**1**	**18386**	**15775**
number frogs * diagnostic group	0.13 (0.14)	-0.15	0.40	1	33957	15039
diagnostic group * age	0.03 (0.20)	-0.37	0.42	1	18327	16680
L2 richness * number frogs * diagnostic group	-0.10 (0.13)	-0.36	0.16	1	32602	16908

Supplementary Material 5 Task 4 (Working memory)

Table A5.1
Model comparisons for Task 4

	elpd_diff	se_diff	elpd_loo	se_elpd_loo	p_loo	se_p_loo	looic	se_looic
**EA-proficiency-model**	0.0	0.0	-1372.3	32.1	207.9	5.8	2744.6	64.6
**EA-richness-model**	-1.4	1.4	-1373.7	32.3	206.8	5.8	2747.4	64.6
**EA-quantity-model**	-3.3	1.9	-1375.6	32.4	208.4	5.8	2751.2	64.8
**ACH-model**	-5.4	2.6	-1377.7	32.7	214.7	6.1	2755.4	66.8

Note. elpd_diff: difference in expected log predictive density (ELPD) between each model and the best-performing model (higher value indicates better predictive performance); se_diff: standard error of the ELPD difference, reflecting uncertainty in the comparison; elpd_loo: expected log predictive density for each model, estimated using LOO; e_elpd_loo: standard error of the ELPD estimate, indicating the uncertainty in model predictive performance; p_loo: effective number of parameters in each model, reflecting model complexity; se_p_loo: standard error of the effective number of parameters; looic: LOO; se_looic: standard error of the LOOIC estimate.

Posterior predictive checks

The posterior predictive checks indicated that the Model (3c) produced plausible predictions, suggesting a good fit to the observed data; see Figure A5.1.

Figure A5.1
Posterior predictive check for the best model of WM

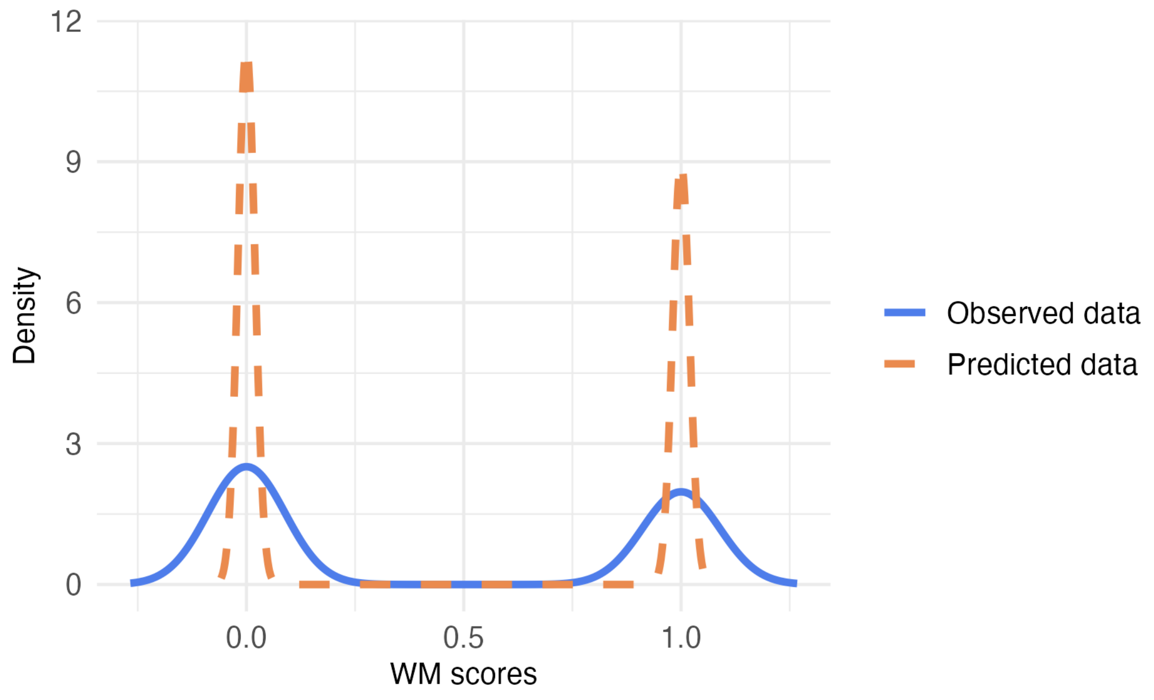

Table A5.2

Result of Model (3c) of working memory

	Estimate (SE)	l-95% CI	u-95% CI	Rhat	Bulk ESS	Tail ESS
**Intercept**	**-0.79 (0.11)**	**-1.01**	**-0.58**	**1**	**12129**	**14102**
**L2 proficiency**	**0.28 (0.11)**	**0.07**	**0.49**	**1**	**11190**	**14415**
**number frogs**	**-1.51 (0.07)**	**-1.65**	**-1.37**	**1**	**23562**	**16912**
**diagnostic group (NT vs ASD)**	**-0.90 (0.25)**	**-1.40**	**-0.42**	**1**	**11677**	**13575**
SES	-0.04 (0.10)	-0.24	0.16	1	11907	14569
**age**	**1.44 (0.12)**	**1.21**	**1.68**	**1**	**12059**	**15260**
attention	0.05 (0.11)	-0.16	0.27	1	11448	14522
IQ	0.48 (0.10)	0.29	0.67	1	11468	14416
L2 proficiency * number frogs	0.08 (0.06)	-0.04	0.21	1	26106	16666
L2 proficiency * diagnostic group	0.38 (0.21)	-0.02	0.79	1	10252	13731
number frogs * diagnostic group	0.17 (0.13)	-0.09	0.43	1	28575	16486
diagnostic group * age	-0.18 (0.24)	-0.65	0.29	1	12009	14285
L2 proficiency * number frogs * diagnostic group	0.18 (0.12)	-0.07	0.42	1	29241	17807

Supplementary Material 6 Shifting

Table A6.1
Model comparisons for Task 5

	elpd_diff	se_diff	elpd_loo	se_elpd_loo	p_loo	se_p_loo	looic	se_looic
**EA-proficiency-model**	0.0	0.0	-3544.5	77.7	208.8	6.8	7089.0	155.5
**EA-richness-model**	0.0	0.7	-3544.6	77.7	208.5	6.8	7089.1	155.5
**EA-quantity-model**	-0.4	0.4	-3544.9	77.8	209.3	6.8	7089.9	155.5
**ACH-model**	-1.5	0.9	-3546.0	77.8	211.3	6.9	7092.0	155.7

Note. elpd_diff: difference in expected log predictive density (ELPD) between each model and the best-performing model (higher value indicates better predictive performance); se_diff: standard error of the ELPD difference, reflecting uncertainty in the comparison; elpd_loo: expected log predictive density for each model, estimated using LOO; e_elpd_loo: standard error of the ELPD estimate, indicating the uncertainty in model predictive performance; p_loo: effective number of parameters in each model, reflecting model complexity; se_p_loo: standard error of the effective number of parameters; looic: LOO; se_looic: standard error of the LOOIC estimate.

Posterior predictive check

Posterior predictive checks indicated that Model (3c) reliably replicated the observed data; see Figure A6.1.

Figure A6.1
Posterior predictive check for the best model of shifting

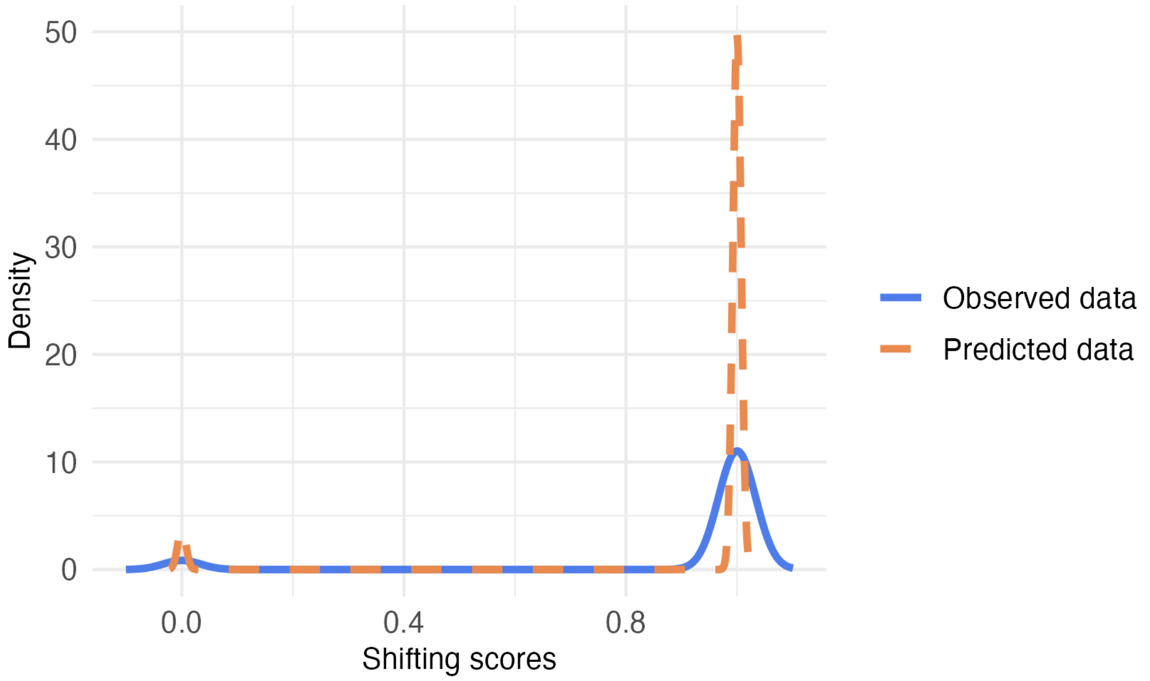

Table A6.2

Result of Model (3c) of shifting

	Estimate (SE)	l-95% CI	u-95% CI	Rhat	Bulk ESS	Tail ESS
**Intercept**	**3.03 (0.08)**	**2.87**	**3.19**	**1**	**9594**	**11981**
L2 proficiency	0.03 (0.09)	-0.13	0.20	1	8583	11452
diagnostic group (NT vs ASD)	0.04 (0.19)	-0.32	0.41	1	8407	12014
SES	0.00 (0.07)	-0.14	0.15	1	8281	12482
**age**	**0.45 (0.09)**	**0.28**	**0.62**	**1**	**8870**	**11945**
attention	0.13 (0.09)	-0.04	0.30	1	8257	12643
IQ	0.11 (0.07)	-0.03	0.25	1	8767	12480
L2 proficiency * diagnostic group	0.13 (0.16)	-0.19	0.45	1	9000	12897
diagnostic group * age	0.05 (0.18)	-0.30	0.39	1	7742	11813

Supplementary Material 7 Switching

Table A7.1

Model comparisons for Task 6

	elpd_diff	se_diff	elpd_loo	se_elpd_loo	p_loo	se_p_loo	looic	se_looic
**EA-proficiency-model**	0.0	0.0	-1531.4	36.7	119.3	3.3	3062.7	73.4
**EA-richness-model**	-3.9	2.7	-1535.3	36.7	118.7	3.2	3070.5	73.5
**EA-quantity-model**	-11.7	5.0	-1543.5	37.1	118.7	3.3	3086.9	74.1
**ACH-model**	-16.6	4.9	-1548.1	37.6	125.2	3.7	3096.3	75.1

Note. elpd_diff: difference in expected log predictive density (ELPD) between each model and the best-performing model (higher value indicates better predictive performance); se_diff: standard error of the ELPD difference, reflecting uncertainty in the comparison; elpd_loo: expected log predictive density for each model, estimated using LOO; e_elpd_loo: standard error of the ELPD estimate, indicating the uncertainty in model predictive performance; p_loo: effective number of parameters in each model, reflecting model complexity; se_p_loo: standard error of the effective number of parameters; looic: LOO; se_looic: standard error of the LOOIC estimate.

Posterior predictive checks

Posterior predictive checks indicated that Model (3c) reliably replicated the observed data; see Figure A7.1.

Figure A7.1

Posterior predictive check for the best model for switching

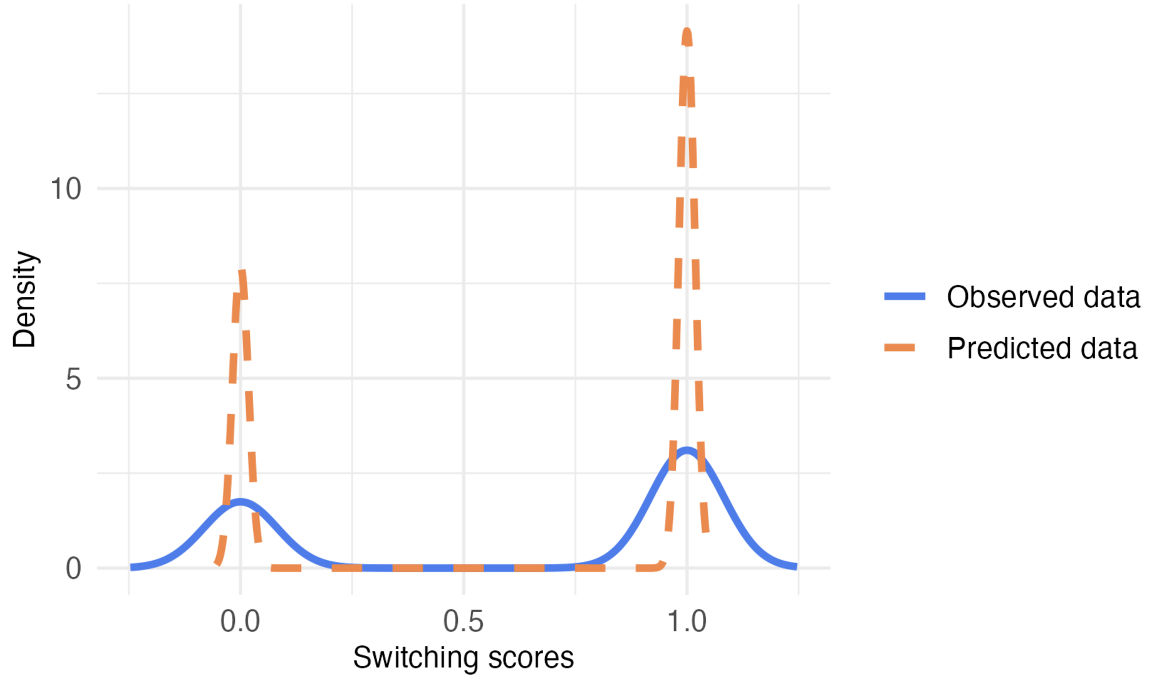

Table A7.2

Result of Model (3c) of switching

	Estimate (SE)	l-95% CI	u-95% CI	Rhat	Bulk ESS	Tail ESS
**Intercept**	**1.29 (0.09)**	**1.11**	**1.47**	**1**	**19837**	**16016**
**L2 proficiency**	**0.19 (0.09)**	**0.01**	**0.36**	**1**	**18204**	**16783**
diagnostic group (NT vs ASD)	-0.16 (0.19)	-0.56	0.24	1	19296	16001
**type (control vs test)**	**-4.37 (0.15)**	**-4.69**	**-4.07**	**1**	**23328**	**15205**
SES	-0.06 (0.06)	-0.19	0.08	1	19250	16719
**age**	**-0.26 (0.07)**	**-0.41**	**-0.10**	**1**	**17734**	**16528**
attention	-0.03 (0.07)	-0.19	0.13	1	20060	16827
IQ	-0.13 (0.07)	-0.26	0.00	1	21241	17052
L2 proficiency * diagnostic group	-0.10 (0.18)	-0.44	0.25	1	18468	16466
**L2 proficiency * type**	**-0.75 (0.15)**	**-1.04**	**-0.26**	**1**	**23292**	**16514**
diagnostic group * type	-0.00 (0.30)	-0.60	0.57	1	28738	15589
diagnostic group * age	0.17 (0.16)	-0.13	0.49	1	18259	16100
L2 proficiency * diagnostic group * type	-0.19 (0.30)	-0.77	0.38	1	24753	15931
